# Primary Follicle Paces Fish Ovarian Maturation Developmental Progression via the Enhancement of Notch and mTOR

**DOI:** 10.3390/biology14121752

**Published:** 2025-12-06

**Authors:** Guangjing Zhang, Xiudan Yuan, Wen Fu, Yujiao Wang, Zhen Huang, Liangyue Peng, Jinhui Liu, Wenbin Liu, Yamei Xiao

**Affiliations:** 1 College of Life Sciences, Hunan Normal University, Changsha 410081, China; kangjing0104@163.com (G.Z.); yuanxd2024@163.com (X.Y.); fuwen@hunnu.edu.cn (W.F.); 18738848447@163.com (Y.W.); 242101020@csu.edu.cn (Z.H.); ply@hunnu.edu.cn (L.P.); jinhuiliu0731@hunnu.edu.cn (J.L.); wenbinliu@hunnu.edu.cn (W.L.); 2 Henan Academy of Fishery Sciences, Zhengzhou 450044, China; 3 Hunan Fisheries Research Institute and Aquatic Products Seed Stock Station, Hunan Academy of Agricultural Sciences, Changsha 410153, China; 4 Yuelushan Laboratory Aquatic Variety Breeding Center, Hunan Normal University, Changsha 410081, China

**Keywords:** primary follicle, ovarian maturation, genomics sequencing Notch/mTOR, fish

## Abstract

Dynamic developmental states of follicles are regarded to be determinants of sexual maturation in fish ovaries. However, it is still a challenge to identify the critical points at which the developmental processes of different types of follicles interact and affect the ovarian development. In this study, four subtypes of the primary follicle (PF) in the ovarian folliculogenesis of zebrafish, i.e., the so-called PF-i, PF-ii, PF-iii, and PF-iv, are first identified by discontinuous NaCl-Percoll gradient centrifugation, as well as their respective morphological features. Then, for the four subtypes of PFs, stage-specific comparative analysis is employed to identify the differentially expressed genes and the differentially methylated regions, which have been validated to be significantly enriched in biological processes encompassing ribosomal biogenesis, meiotic progression, transcriptional regulation, and mitochondrial respiration. Results from transcriptional analysis further demonstrate significant changes in the expression profiles at different developmental stages from the PF-ii to the PF-iii. By molecular biology identification, it is shown that the enhancement of Notch and mTOR pathways can significantly regulate the ovarian development through the pacing effect of primary follicles. Clearly, all these uncovered results could provide a deeper understanding of the initial regulation of ovarian maturation, as well as a new multidisciplinary analytic tool to study follicle candidate regulators in the developmental process of other fish.

## Simple Summary

Ovarian follicles, as the fundamental structural and functional units of the ovaries, undergo a complex process of molecular regulation during development. Zebrafish often serve as an excellent model organism for the investigation of organogenesis and developmental regulation, basically in virtue of their small size, short reproductive cycle, and high fecundity. In this study, the zebrafish are employed to isolate and purify primordial follicles (PFs) so as to identify their heterogeneity. Through integrated multi-omics analysis, we decipher the molecular regulatory pathways underlying the PF development, together with the identification of the related key genes and signaling pathways. Clearly, the uncovered cellular features in ovarian development and the developmental dynamics of zebrafish PFs by this study could provide a theoretical foundation for interventions in fish sexual maturation.

## 1. Introduction

Ovarian development depends on the dynamic state of oogenesis, which refers to the process first from primordial germ cells to oogonia and then from oogonia to a mature ovum [1,2]. After a certain period of proliferation, some oogonia begin to undergo their first meiosis and become primary oocytes. The cell division cycle of primary oocytes is interrupted after reaching the so-called dictyotene stage from the leptonema through the amphitene, pachynema, and diplonema. At this point, the oocyte is surrounded by a layer of granulosa cells (follicular cells), which first form primordial follicles and subsequently form primary follicles (PFs). Afterwards, the follicles enter the growth and development stage, while the oocyte grows and develops inside the follicle until mature ovulation [3,4].

In teleosts, folliculogenesis is generally divided into three stages: primary follicles (PFs), secondary follicles (SFs), and mature follicles (MFs) [5]. In teleosts, the previtellogenic follicle (PF) stage, also known as the perinucleolus stage of primary oocyte development, corresponds to the slow growth phase of folliculogenesis. During embryonic development, germ cells activate the expression of meiosis-associated genes, undergo premeiotic DNA replication, and initiate meiosis, establishing the developmental basis for oocyte growth at the PF stage [6,7,8]. Cortical alveoli were synthesized before or concomitantly with the endocytosis of lipids and vitellogenin, which was indicative of the initiation of PFs transition to SFs [8,9]. The SFs are characterized by the accumulation of yolk granules in oocytes, with a well-developed radial band and two distinct strata of follicular cells [6,10]. In teleosts, the onset of puberty in females was marked by the appearance of the first wave of pre-vitellogenic stage follicles from the PFs stage [11,12,13,14]. It reported that the older the age of initial sexual maturity, the longer the duration of the PFs stage [15,16,17,18,19,20]. In addition, the key change in folliculogenesis in the delayed puberty of artificially domesticated fish lies in the prolonged PFs stage [17,18,21].

Despite extensive work on vitellogenesis, meiotic resumption, and late follicle maturation, the early follicular phase, particularly the developmental heterogeneity within PFs and its impacts on ovarian maturation, remains poorly characterized [22]. Among the important issues that need to be addressed, it is valuable to answer whether the PFs comprise distinct subtypes with different developmental potentials or not, and how the epigenetic and transcriptional programs within the PFs affect the timing and pace of follicle progression.

In this paper, we aim to study the developmental characteristics of PFs in fish and their possible rate-limiting effects on ovarian follicle development. With zebrafish being the research model, we will first conduct the NaCl-Percoll density gradient centrifugation so as to reveal potential subtypes of PFs. Then, by integrating analysis of methylation sequencing and Transcriptome sequencing, we perform stage-by-stage comparative studies among the four subtypes of PFs and construct the profiles of differentially methylated sites and transcriptional expression. Then, clustering and enrichment analyses are further employed to reveal the dynamic expression patterns of differentially expressed genes and screen the related key signaling pathways in the PFs development.

## 2. Materials and Methods

### 2.1. Fish

Zebrafish were maintained at the Engineering Research Center of Ployploid Fish Reproduction and Breeding of the State Education Ministry, Hunan Normal University.

### 2.2. Ethics Statement

Fish work was performed in strict accordance with the recommendations in the Guidelines for the Care and Use of Laboratory Animals of the National Advisory Committee for Laboratory Animal Research in China and was approved by the Animal Care Committee of Hunan Normal University (Permit Number. 4237).

### 2.3. Density Gradient Separation of Primary Follicles

Sexually mature zebrafish were chosen as the research subjects due to the presence of ovarian follicles at various developmental stages in their ovaries [4]. Ovarian tissues of zebrafish (10–15) were collected after anesthesia with laden fish stabilizer medicine, then digestion with 1 mg/mL Collagenase I and DNAase I (EN0521, Thermo Fisher Scientific, Waltham, MA, USA) was added at room temperature for 20–25 min. The digestion was terminated with D-Hanks solution and centrifuged at 800 rpm for 10 min. The supernatant was discarded and then cleaned with D-Hanks solution. The mature follicles and large secondary follicles were filtered using a 100-mesh cell strainer [23], and the remaining mixed follicles were reserved for use.

The mother liquor was prepared with a 100% Percoll solution (Solarbio, Beijing, China) with 1.5 mM NaCl at 9:1. In the experiment, each concentration of Percoll separation liquid was used with 0.15 mM NaCl diluted into 20%, 25%, 30%, 35%, 40%, 45%, 50%, and 60% Percoll liquid (determined by pre-experiment). The collected mixed follicles were carefully laid along the tube wall on top of the density gradient solution, stood for 10–15 min, and then were centrifuged at 800 rpm for 10 min. Follicles with different density gradients were carefully collected by pipette and separately placed in a 35 mm Petri dish (Figure S1). Fiji (Fiji Is Just ImageJ), version 2.9.0 was used to measure the diameter of the obtained follicles. The number of samples measured corresponded to the total number of follicles counted across five distinct visual fields, with the experiment being repeated multiple times to ensure the reliability and consistency of the results.

### 2.4. Histological, Transmission Electron Microscope (TEM), Immunohistochemistry, and Immunofluorescence

Ovarian tissues or follicles were fixed in the paraformaldehyde solution. The fixed samples were dehydrated and embedded in paraffin. Sections were cut at 5~6 µm using a Leica RM2015 Microtome (Leica Microsystems, Wetzlar, Germany) and then transferred to slides, which were processed for hematoxylin and eosin staining [24]. For size measurements and quantitative statistics of follicles in ovarian tissue, five skip-cut sections from each sample were analyzed, and 5~10 fields were selected in each section. Each stage fish had 3 parallel samples.

The follicular precipitates of different sizes were collected in 1.5 mL EP tubes and fixed with 3% glutaraldehyde solution. After alcohol dehydration, acetone infiltration, epoxy resin embedding, ultrathin sectioning with an EM UC6 machine (Leica Microsystems, Vienna, Austria), and uranyl acetate lead citrate staining, the sections were observed and photographed using a transmission electron microscope (Japanese JEM-1230, JEOL Ltd., Tokyo, Japan).

Preparation of sections required for immunohistochemistry (IHC) and immunofluorescence (IF). Ovary tissues were fixed in 4% paraformaldehyde, dehydrated, embedded in paraffin, and then sliced and dewaxed with xylene. Next, microwave antigen repair was performed using the citrate antigen retrieval solution (E673000-0100, Sangon Biotech, Shanghai, China).

For the IHC assay, the slices were soaked in 3% H_2_O_2_ at room temperature for 30 min away from the light and washed in Tris-buffered saline with Tween-20 (TBST) 3 times. Then, they were blocked with 10% serum from the same source as the primary antibodies at room temperature for 30 min. The primary antibodies were added and incubated at 4 °C overnight; the primary antibodies used were mTOR (HA500126, Huabio, Hangzhou, China) and Notch (AF5296, Affinity Biosciences, Cincinnati, OH, USA). The secondary antibodies anti-rabbit IgG (1:500) (ab150113, Abcam, Cambridge, UK) were added and incubated at room temperature for 1 h. Wash on TBST 3 times. Diaminobenzidine (DAB) working solution was added to the slide, and dyeing was terminated with water according to the degree of dyeing. The nucleus was stained with DAPI solution for 10 min. For the IF assay [25], the samples were blocked with 2% bovine serum albumin (BSA) at room temperature for 30 min. Overnight incubation of the primary antibodies at 4 °C, the primary antibodies used synaptonemal complex protein 3 (Sycp3) (1:200) (provided by the laboratory of Wuhan Institute of Water Science, Wuhan, China). TBST was washed 3 times. The secondary antibodies anti-mouse IgG (1:500) were added and incubated at room temperature for 1 h and stained with DAPI solution for 10 min. Fluorescence was imaged using a Zeiss LSM510 confocal microscope (Carl Zeiss AG, Oberkochen, Germany).

### 2.5. Chromosome Spreading

The follicle group collected in the EP tube was washed 3 times with PBS. A 0.075 mol/L KCl solution (3× volume) was added, and the mixture was gently mixed before being incubated for 2 h at room temperature (hypotonic treatment). After incubation, 4% paraformaldehyde (1/3 volume) was added for fixation. Samples (60–100 μL) were then applied to slides and air-dried for 1–2 h at 4 °C in the dark. Following drying, slides were incubated with synaptonemal complex protein (Sycp1) and Sycp3 antibodies (provided by the laboratory of Wuhan Institute of Water Science, Wuhan, China). Fluorescence was imaged using a Zeiss LSM510 confocal microscope (Carl Zeiss AG, Oberkochen, Germany).

### 2.6. Staining Nucleolar Organizer Region

The ovarian tissue was sliced and dewaxed in xylene. Then, the slice was immersed in Nucleolus composition region silver staining solution kit (AgNOR stain) solution (DK0047, LEAGENE, Beijing, China)) and was incubated at room temperature for 40–60 min. The TBST was cleaned 3 times.

### 2.7. DiOC6 and DAPI Staining

The ovarian tissue was sliced and dewaxed in xylene. The sections were incubated with the DiOC6 (Solarbio, Beijing, China) probe for 15 min in the dark, and then they were cleaned with TBST. The sections were stained with DAPI for 10 min. Then, fluorescence was imaged using a fluorescence microscope.

The four subtypes of PFs were stained with DAPI for 10 min, and fluorescence imaging was performed using a fluorescence microscope. ImageJ software was utilized to estimate the number of follicular cells surrounding each intact follicle. For each type of PF, the sample size was *n* ≥ 50.

### 2.8. RNA-Sequencing

Three independent biological replicates were used in each subtype of PFs. Illumina sequencing was carried out by pooling the libraries according to the requirements of effective concentration and target data volume at Beijing Novogene Company, Beijing, China. Use the HISAT2v2.0.5 software to build the reference genome index for zebrafish (https://www.ncbi.nlm.nih.gov/datasets/genome/GCF_000002035.6/, GRCz11) (accessed on 4 December 2023). The FPKM value of each gene was calculated according to the length of the gene and was used as the final expression amount of the gene. The significant substantial difference was defined if |log2(FoldChange)| > 1 and *p*-value < 0.05 (both thresholds were met simultaneously).

The Cluster Profiler R software package (Cluster GVis, Version 4.3.2) was used for gene ontology (GO) enrichment and KEGG pathway statistical enrichment analysis of differentially expressed genes (DEGs), and the gene length deviation was corrected. After correction, the items with *p*-value < 0.05 were considered to be significantly enriched by differentially expressed genes. Weighted correlation network analysis (WGCNA) was conducted in the NovoMagic (https://magic.novogene.com/customer/main#/loginNew) (accessed on 18 May 2024). The Pearson correlation algorithm was used to calculate the correlation coefficient and *p*-value of module characteristic genes and traits. The correlation between modular gene expression and corresponding traits was calculated for each trait-related module. GO enrichment and KEGG pathway statistical enrichment analysis were performed on the module genes.

### 2.9. Methylation Sequencing and Analysis

Three independent biological replicates were used in each subtype of PF. Illumina sequencing was carried out by pooling the libraries according to the requirements of effective concentration and target data volume at Beijing Novogene Company, Beijing, China. Double-ended sequencing analysis was performed using the Illumina sequencing platform. The Bismark software (version: 0.23.1) was used to compare the zebrafish genome (GCF_00000203.5). Use the software cgmaptools (version: 0.1.1). Methylation correlation analysis was performed (C-base coverage statistics, C-base sequencing depth, average C-base methylation ratio, average C-base methylation level, different types of methylation ratios, different types of methylation levels, and DNA methylation distribution in gene regions).

DMR detection uses the cgmaptools software published by authoritative journals (version: 0.1.1). The differential methylation region was obtained, and the differential DMR interval was determined according to the selected interval of the two samples, with thresholds of P-value ≤ 0.001 and DMR ≥ 0.2. Upregulation of statistical CG, CHG, and CHH methylation (C base distribution on the genome contains three forms of CG, CHG, and CHH, where H represents either A or T, or C bases). GO analysis was performed for differentially methylated genes involved in major biological functions, and KEGG analysis was further performed to analyze the signaling pathways involved in enrichment in these genes (*p* < 0.05 was considered a significant difference).

### 2.10. Quantitative Real-Time PCR (qRT-PCR)

For each treatment group, three zebrafish were selected. Following anesthesia with a fish stabilizer-containing solution, ovarian tissues were collected and lysed using TRIzol reagent (Takara Bio Inc., Kusatsu, Shiga, Japan). Total RNA was then extracted with a commercial RNA extraction kit (Magen, Guangzhou, China) and subsequently reverse-transcribed into cDNA with the TAKARA reverse transcription kit (Takara Bio Inc., Kusatsu, Shiga, Japan) according to the manufacturer’s instructions. The primers were designed using the NCBI Premier (Primer designing tool (nih.gov)), and the presence of dimers was tested with Olige 7 software, and the primers were synthesized by Qingke Bio, Beijing, China (Supplemental Table S1). The reference gene for the qRT-PCR was *β-actin*, and the analytical instrument was Quantstudio 5 (Thermo Fisher Scientific, Waltham, MA, USA). The results were calculated using the 2^−ΔΔCT^ method. For the mRNA expression data in four follicle subtypes, differences among groups were analyzed by one-way analysis of variance (one-way ANOVA) followed by Tukey’s honest significant difference (HSD) post hoc test for pairwise comparisons. For the mRNA expression data in three groups: control and two treatments, one-way ANOVA was used to assess overall group differences, and Dunnett’s post hoc test was subsequently employed to compare each treatment group with the control group. A *p* < 0.05 was considered statistically significant.

### 2.11. Inhibitor Treatment and Added Amino Acid Feeding

The Notch signaling pathway inhibitor Inhibitor of Mastermind Recruitment-1 (IMR-1) [26,27] (Selleck, S8280, Selleck Chemicals, Houston, TX, USA) and the mTOR signaling pathway inhibitor Palomid 529 (P529) [28,29] (Selleck, S2238) were separately dissolved in DMSO to prepare their 10 mM stock solutions. The optimal treated concentrations of IMR-1 (1 µM and 5 µM) and P529 (1 µM and 5 µM) were determined through preliminary experiments. Zebrafish fry at 25 days post-hatching (dph) were treated with the inhibitor. After 35 days of treatment, 3–5 zebrafish from each group were sampled to examine the histological changes in their ovaries.

Dietary lysine supplementation can also improve production and reproductive performance. DL-methionine (DL-Met) and L-lysine hydrochloride (L-Lys·HCl) were commonly added to feed [30,31,32]. Different amounts of DL-Met (K_4_O_7_P_2_, Anhui Zhonghong Bioengineering Co., Ltd., Hefei, China) and L-Lys·HCl (211-519-9, Anhui Zhonghong Bioengineering Co., Ltd., Hefei, China) were added to the zebrafish diet. The specific proportions of added amino acids were determined through preliminary experiments, and for the experimental groups, DL-Met^2.5%^/L-Lys·HCl^7.5%^ and DL-Met^5.0%^/L-Lys·HCl^7.5%^ were selected. Zebrafish fry at 25 dph were added with DL-Met^+^/L-Lys·HCl^+^. After 2 weeks of adding, 3–5 zebrafish from each group were collected to observe the histological changes in their ovaries.

### 2.12. Western Blot

Ovaries were collected from zebrafish in the control group, inhibitor-treated group, and amino acid–supplemented feed group (n = 3 per group). Total proteins were extracted using a commercial protein extraction kit (Thermo Fisher Scientific, Waltham, MA, USA), and protein concentrations were determined with a BCA assay (Beyotime, Shanghai, China) [33]. Appropriate concentrations of sodium dodecyl sulfate-polyacrylamide gels were prepared for electrophoresis. After loading the protein samples, constant pressure electrophoresis was carried out, and the proteins were then transferred to the nitrocellulose membrane.

Membranes were blocked with 3–5% skim milk for 1.5 h at room temperature, followed by overnight incubation at 4 °C with the following rabbit monoclonal primary antibodies: Notch (1:1000, AF5296, Affinity Biosciences, Cincinnati, OH, USA), mTOR (1:1000, HA500126, Huabio, Hangzhou, China), p-mTOR (1:1000, HA721632, Huabio), S6K1 (1:500, HA500095, Huabio), p-S6K1 (1:1000, 9205S, Cell Signaling Technology, Danvers, MA, USA), α-tubulin (1:1000, ER130905, Huabio), and β-actin (1:1000, EM21002, Huabio). After three washes in TBST, membranes were incubated with HRP-conjugated anti-rabbit secondary antibody (1:1000) for 1–2 h at room temperature. Chemiluminescence signals were captured using the Bio-Rad ChemiDoc MP Imaging System (Bio-Rad Laboratories, Hercules, CA, USA).

For the protein blot results of the ovarian samples from the control group and the treatment group (with n = 3 in each group), the Average Optical Density (AOD) was calculated based on the mean optical density of the positive signals using the ImageJ software. The data were first validated to meet the assumptions of normality (Shapiro–Wilk test) and homogeneity of variance (Levene’s test). Differences in protein expression among the three groups (control and two treatments) were analyzed by one-way ANOVA. To identify specific differences between the control group and each treatment group, Dunnett’s post hoc test was performed after ANOVA. Statistical significance was defined as *p* < 0.05.

## 3. Results

### 3.1. Classification of Primary Follicles and Their Cytological Characteristics in Zebrafish

The mixed follicles, filtered by a 100-mesh cell strainer, could be enriched in 5 groups through discontinuous NaCl-Percoll gradient centrifugation separation (Figure S1A–C). The follicles of Groups 1–4 were PFs, in which monolayer follicular cells tightly wrapped around the oocyte; however, the Group 5 follicles were the SFs with cortical alveoli in the oocytes and around bilayer follicular cells (Figure S1B–D).

The follicles in Groups 1–4 were named PF-i, PF-ii, PF-iii, and PF-iv, respectively. Among them, the PF-i were uniformly clustered in the 20% and 25% Percolls, with a mean diameter of 23.0 μM (12.6~36.2 μM). The PF-ii were clustered in the 30% Percoll, with a mean diameter of 64.3 μM (34.4~82.8 μM). The PF-iii were clustered in the 35% and 40% Percoll, with a mean diameter of 88.9 μM (60.120~116.825 μM). The PF-iv were clustered in the 45% to 50% Percoll, with a diameter of 111.4 μM (80.895~128.063 μM) (Figure S1B–D, Table S2).

**Figure 1:**
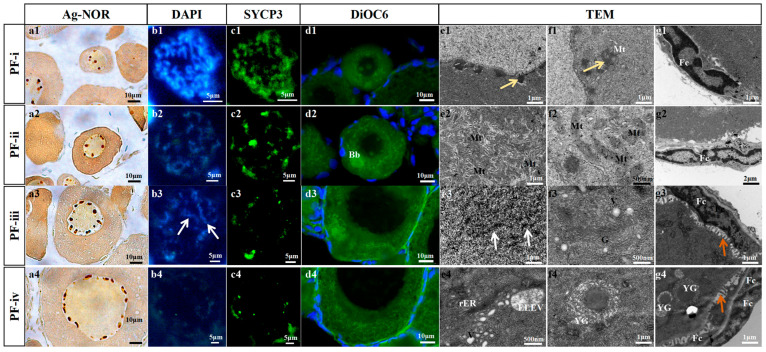
**Cytological observation of four subtypes of PFs in zebrafish.** (**a1**~**a4**) Nucleolus observation of different types of PFs using Ag-NOR staining. (**b1**~**b4**,**c1**~**c4**) Chromosomal observation of different types of PFs immunostained with anti-Sycp3 (green) antibody and stained with DAPI. (**d1**~**d4**) Mitochondria observation of different types of PFs by DiOC6 (membrane marker of organelles) and DAPI (blue). (**e1**~**e4**,**f1**~**f4**,**g1**~**g4**) Transmission Electron Microscopy (TEM) ultrastructure observation of different types of PFs. Fc: follicle cell; Mt: mitochondria; Bb: Balbiani body; G: Golgi apparatus; V: vesicles; rER: rough endoplasmic reticulum. ELVE: EndoLysosomal Vesicular Assemblies; YG: yolk granule. The white arrows refer to the lampbrush chromosome, the yellow arrows show electron-dense clusters, and the orange arrows show microvilli.

In the oocyte of PF-i, 3–5 nucleoli were observed in the cross-section, and its chromosomes were tight with high expression levels of synaptonemal complex protein 3 (Sycp3) and synaptonemal complex protein 1 (Sycp1) (Figure 1(a1,b1,c1) and Figure S2). In addition to the diplotene of the meiosis I prophase, the zygotene and pachytene were observed in the oocytes of PF-i (Figure S2). The result of DiOC6 probe detection showed that mitochondria were distributed in the PF-i oocyte cytoplasm adjacent to the nucleus (Figure 1(d1)). Dense clusters were observed outside the oocyte nucleus of PF-i; some of them were surrounded by spherical mitochondria (Figure 1(e1,f1)). A total of 18 ± 5 follicular cells were tightly wrapped around the oocyte in PF-i (Figure 1(g1) and Figure S3).

In the PF-ii, the nucleoli of the oocyte increased to 8~10 (Figure 1(a2)). The crossover phenomenon was observed in the homologous chromosomes of the PF-ii oocyte, and the expression level of Sycp3 was down-regulated (Figure 1(b2,c2)). Abundant mitochondria gathered on one side of the cytoplasm of oocytes, forming the Balbiani body (Figure 1(d2,e2)). Megamitochondria and abundant endoplasmic reticulum appeared in the oocyte cytoplasm of this subtype (Figure 1(f2)). A monolayer of 90 ± 20 follicular cells surrounded the oocyte in PF-ii, and the extracellular matrix associated with the oocyte began to thicken (Figure 1(g2) and Figure S3).

For the PF-iii, there were 15~20 nucleoli in their oocyte cross-section (Figure 1(a3)). Sycp3 was weakly expressed on the oocytes, and the lampbrush chromosomes were observed (Figure 1(b3,c3,e3)). The aggregated mitochondria began to disperse, but the Golgi complex and its secreted vesicles increased significantly in oocytes (Figure 1(d3,f3)). The number of single-layer follicular cells outside the oocyte increased to 186 ± 21, and microvilli appeared (Figure 1(g3), Supplemental Figure S3).

In the oocyte of PF-iv, there was an extremely weak expression of Sycp3 (Figure 1(a4,b4,c4)), and a large number of mitochondria and vesicle droplets were observed (Figure 1(d4,e4)). Bits of intracellular yolk granules were present (Figure 1(f4)). Bilayer follicular cells (360 ± 33) and zona radiata appeared in the local area of PF-iv (Figure 1(g4) and Figure S3).

### 3.2. Gene Expression Profiles of Four Subtypes of PFs

**Figure 2:**
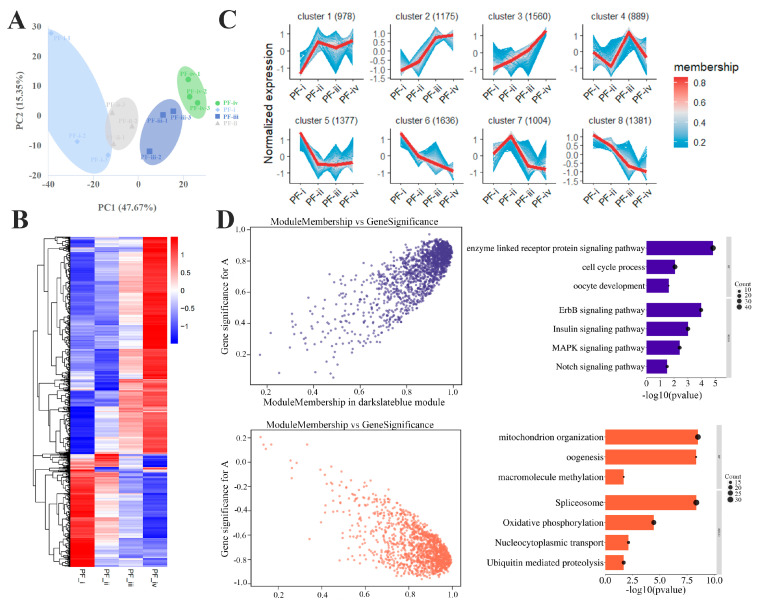
**Transcriptomic analysis of different types of PFs in zebrafish.** (**A**) Principal component analysis of Transcriptome. (**B**) Differential gene clustering heat map. (**C**) Divided into 8 clusters according to gene expression patterns. (**D**) Two gene expression clusters correlate highly with PF development and their functional enrichment (GO and KEGG) in WGCNA.

Gene expression profiles of the four subtypes of PFs (PF-i, PF-ii, PF-iii, and PF-iv) in zebrafish were analyzed by the RNA-Seq technique. Performing principal component analysis (PCA), it was found that each sample had high repeatability and the farthest distance between the PF-ii and PF-iii compared to the other two adjacent stages (PF-i and PF-ii, PF-iii and PF-iv) (Figure 2A). The differential gene heat map further indicated that the expression profiles significantly changed from the PF-ii to PF-iii (Figure 2B).

Based on the top 10,000 genes with FPKM value, we obtained eight clusters about the dynamic change trend of gene expression in the four subtypes through the getClusters function (Figure 2C). As shown in Supplemental Figure S4A, the highly expressed genes in the PF-i (Cluster 5) were mainly involved in DNA metabolism and methylation and nucleoplasmic material transport. Functional enrichment of highly expressed genes in the PF-ii (Cluster 7) was shown in ribosome biogenesis and rRNA metabolism. In the PF-iii, the highly expressed genes (Cluster 4) were involved in activating the biosynthesis of macromolecules, such as nucleoplasmic transport and DNA-templated transcription. In Cluster 2, the expression of many maternal zygote genes (associated with organ development: heart, eye, trunk) and the Notch signaling pathway increased significantly at the PF-iii and PF-iv stages.

Two highly coexpressed gene clusters related to oocyte development were identified in WGCNA. The genes in one cluster (dark slate blue module) were positively correlated with oocyte growth (r = 0.85; *p* < 0.001), such as the Notch and Insulin signaling pathways. The genes in another cluster (coral1 module) were negatively correlated with oocyte size (r = −0.82; *p* < 0.001) associated with ribosome biogenesis, mitochondrial organization, and gene methylation (Figure 2D). It is worth noting that the significant changes in gene expression of these two clusters from the PF-ii to PF-iii (Supplemental Figure S4B).

### 3.3. Analysis of Methylated Region Changes in Four Primary Follicle Subtypes

**Figure 3:**
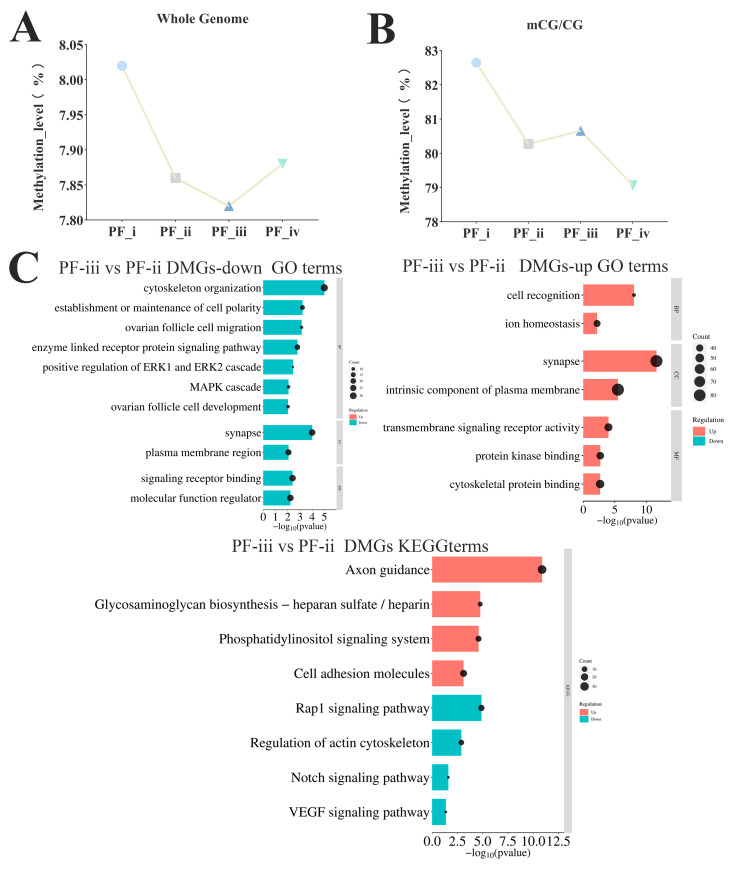
**Transcriptomic and whole-genome methylation analysis of different types of PFs in zebrafish.** (**A**) Whole genome DNA methylation map of PFs in four different subtypes of zebrafish. (**B**) CG DNA methylation of PFs in four different subtypes of zebrafish. (**C**) The GO and KEGG enrichment analysis of differentially methylated sites in the PF-ii to PF-iii stage.

Whole-genome DNA methylation sequencing was performed for different subtypes of PFs (PF-i, PF-ii, PF-iii, and PF-iv, 3 replicates per sample) in zebrafish using the WGBS technique. An average of 227 million to 257 million clean reads were generated in the 12 samples after data filtering, and 66.42% to 69.06% of the total reads were compared to the reference genome (genome assembly GRCz11). The number of CpG sites covered by CpGs with a depth greater than or equal to 5× was 139–191 million methylation sites (Tables S3 and S4). The highest level of whole genome methylation occurred in the PF-i stage, demethylation began in the PF-ii stage, and the methylation level decreased to the lowest point in the PF-iii stage, then the methylation level was high again in the PF-iv stage (Figure 3A). Moreover, CpG islands (CGIs) showed different trends among PF-ii, PF-iii, and PF-iv stages, with an increase in methylation levels from PF-ii to PF-iii, but demethylation at PF-iv (Figure 3B).

The results of circos plots showed that the changes in DNA methylation levels of differential methylation regions (DMRs) (*p*-value ≤ 0.001, DMR ≥ 0.2) were significantly more sites with increased methylation levels from the PF-ii to PF-iii stage (Figure S5). Functionally enriched terms in DMRs (PF-ii to PF-iii stage) were mainly involved in biological activities such as the maintenance of cell polarity and cell macromolecular localization, and KEGG results showed that the regions were enriched in the Notch and VEGF signaling pathways (Figure 3C).

### 3.4. Regulatory Effect of Notch/mTOR on the Development of Primary Follicles in Zebrafish

**Figure 4:**
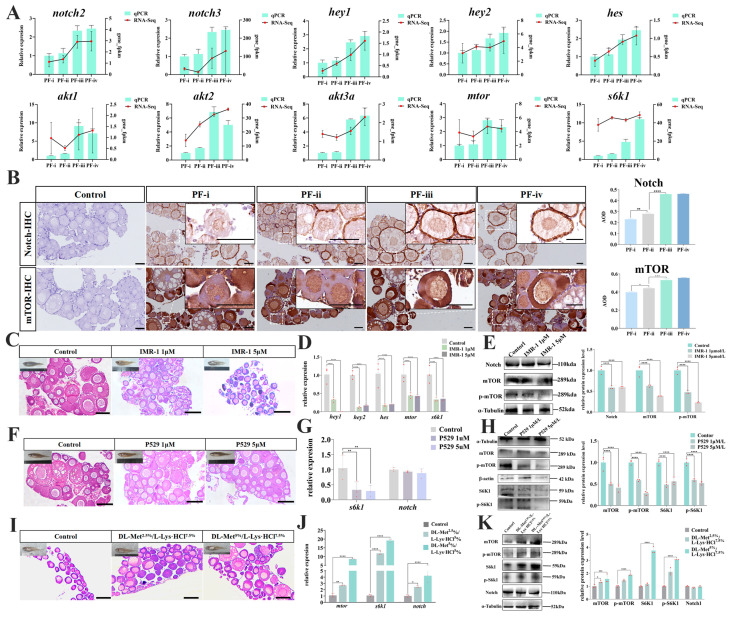
**Regulatory effect of Notch/mTOR on the developmental process of PFs in zebrafish.** (**A**) qRT-PCR to detect the expression of *notch2*, *akt2*, and *mtor* genes in different types of PFs in zebrafish. (**B**) Immunohistochemistry (IHC) to detect the Notch and mTOR proteins localization in the ovary and expression of different types of PFs of zebrafish; scale bar, 50 μM; (**C**,**F**,**I**) Histological observation of the ovary in the treated group with IMR-1, P529, and dietary amino acid supplementation; scale bar, 100 μM. (**D**) The *hey1*, *hey2*, *hes*, *mtor*, and *s6k1* mRNA levels in the ovary tissues of IMR-1-treated groups. (**E**) The Notch, mTOR, and p-mTOR protein levels were expressed in the ovarian tissues of IMR-1-treated groups. (**G**) The *s6k1* and *notch* mRNA levels were expressed in the ovarian tissues of the P529-treated groups. (**H**) The mTOR, p-mTOR, S6K1, and p-S6K1 protein levels of expression in the ovarian tissues of the P529-treated groups. (**J**) The *mtor*, *s6k1*, and *notch* mRNA levels of expression in the ovarian tissues of the amino acid addition group. (**K**) The mTOR, p-mTOR, S6K1, P-S6K1, and Notch protein levels were expressed in the ovarian tissues of the amino acid addition group. “*”: *p* < 0.05; “**”: *p* < 0.01; “***”: *p* < 0.001*;* “****”: *p* < 0.0001. (means ± SD of relative expression; *n* = 3 for each group).

We further conducted integrated analyses of DEG and DMR profiles during this development. There is a positive correlation between DMR-down and RNA-up in PF-ii to PF-iii (Figure S6). Results of the RNA-Seq and the qRT-PCR showed that the mRNA levels of Notch and mTOR signaling pathways (*notch2*, *notch3*, *hey1*, *hey2*, *hes*, *akt1*, *akt2*, *akt3a*, *mtor*, and *s6k1*) were significantly up-regulated from the PF-ii to the PF-iii (Figure 4A). Immunohistochemical detection indicated that Notch protein was predominantly localized on the oocyte membrane, while mTOR was in the cytoplasm of oocytes. The IHC results support the previously mentioned results, where a larger immunomarked area is observed for both Notch and m-Tor in PF-iii and PF-iv (Figure 4B).

To further investigate the effect of Notch on ovarian maturation in fish, zebrafish fry (25 dph) were treated with the Notch inhibitor (Mastermind Recruitment-1, IMR-1). The histological changes in the zebrafish ovary after 35 days owing to inhibitor treatment were shown in Figure 4C. In the 1 μM IMR-1 group, there were three observed subtypes of follicles, i.e., the PF-i, the PF-ii, and the PF-iii. The 5 μM IMR-1 group showed only the PF-i and PF-ii in the ovarian tissue. While there were four PF subtypes and SPs in the ovaries of the control group zebrafish (60 dph) (Figure 4C). Specifically, compared with the control group, the mRNA levels of *notch* downstream target genes *hey1*, *hey2*, and *hes* in the ovary tissue treated by IMR-1 were significantly down-regulated, as well as the expression level of *mtor* and its downstream *s6k1* gene (Figure 4D). Moreover, the protein level of mTOR, especially its phosphorylation level, was more down-regulated than that of the control group (Figure 4E).

Palomid 529 (P529) is an inhibitor of mTOR. In the 1 μM P529 group, four PF subtypes and SFs were observed in the ovarian tissues, where the quantity of PF-iii decreased compared with the control group. Increasing the concentration of P529 (5 μM) could lead to the number of SFs dropping dramatically (Figure 4F). The mRNA levels of *s6k1* genes were significantly down-regulated, while the *notch* mRNA levels remained unchanged in the P529-treated groups (Figure 4G). Moreover, the protein levels of mTOR and S6K1, especially their phosphorylation levels, were more down-regulated than those of the control group (Figure 4H).

We added DL-Met and L-lysine hydrochloride (L-Lys·HCl) (DL-Met^+^/L-Lys·HCl^+^) to the diet of 25 dph zebrafish. After being fed for two weeks, four PF subtypes, even SFs, could be observed in the DL-Met^+^/L-Lys·HCl^+^ groups, while only the PF-i and PF-ii were visible in the ovaries of the control group (Figure 4I). Western blot analysis showed that not only the mRNA levels but also the protein and phosphorylation levels of mTOR and S6K1 in the ovary tissues of the DL-Met^+^/L-Lys·HCl^+^ groups were up-regulated compared with those in the control group. However, the Notch mRNA and the protein levels did not change in the DL-Met^+^/L-Lys·HCl^+^ group (Figure 4J,K).

## 4. Discussion

### 4.1. Isolation and Purification of PFs

The isolation and purification of follicles is one of the essential parts of follicular research [4,34]. Various collagen types and hyaluronic acid were used in isolation protocols for mouse and bovine follicles [35,36]. Yaniv M optimized the use of all collagens in zebrafish and then obtained all stages of oocytes by strainer isolation based on the different sizes of stages of oocytes [4]. However, the sieve separation process could lead to the rupture of oocytes [37]. Percoll is composed of colloidal silica coated with polyvinylpyrrolidone (PVP). Due to its low permeability, non-cell-penetrating property, low viscosity, high density, and non-toxicity, it is an ideal medium [38]. Currently, Percoll density gradient centrifugation has been successfully used to purify various animal cells, including bone marrow cells, red blood cells, white blood cells, lymphocytes, liver cells, primary cardiomyocytes, sperm, etc. [39,40]. In this study, we established a Percoll density gradient system prepared by diluting 100% Percoll with NaCl solution, which reliably separated PFs into distinct density layers (20–50%) and SFs into 60% Percoll. The density gradient system we established can also be used for the isolation of PFs of crucian carp (*Carassius auratus*), *Megalobrama amblycephala,* and grass carp (*Ctenopharyngodon idella*) (results not published). These results demonstrate the robustness and wider utility of this gradient-based method for early follicle isolation.

### 4.2. Refined Classification of PFs

Selman et al. categorized the primary follicle growth stage in zebrafish into two stages based on oocyte morphological criteria and physiological and biochemical events. In Stage Ia, the oocyte resides within the germ cell capsule and undergoes the leptotene and zygotene stages of meiosis I prophase. In stage Ib, the oocyte is enclosed within the follicle by follicular cells, undergoes the pachytene, and is arrested in the late diplotene of the meiotic I prophase [7,41]. Elkouby and Mullins considered the many distinct dynamics during specific early prophase stages and further refined the staging description of early follicular development (Stage I), oogonia (St.Ia^oogonia^; 9–11 μM), and the meiotic prophase stages St.Ia^leptotene^ (8–9 μM), St.Ia^zygotene^ (10–16 μM), St.Ib^pachytene^ (17–19 μM), to St.Ib^diplotene^ (20–140 μM). Balbiani body components polarize around the centrosome in St.Ia^zygotene^ and continue to nucleate within an indentation in the nuclear envelope termed the nuclear cleft during St.Ib^pachytene^ to St.Ib^diplotene^ [4,6,42]. In this study, the PFs were further subdivided into four stages (PF-i, PF-ii, PF-iii, and PF-iv). In PF-i, oocytes were zygotene, pachytene, and diplotene of the meiosis I prophase; Balbiani body components appeared around the nucleus. In the PF-ii stage, oocytes are arrested at meiosis at the diplotene stage with distinct Balbiani body structures localized to the cytoplasmic side. In PF-iii, the appearance of lampbrush chromosomes and rich vesicles in the cytoplasm reveals that active nucleoplasmic communication and RNA transcription activities are experienced. In the PF-iv stage, oocytes enhance communication with the outside world through microvilli and bilayer follicular cell structures. Compared with the Selman stage criteria, we subdivide the late St. Ia stage and St. Ib stage into four stages. Compared with the Elkouby stage criteria, PF-i included the follicles from the St.Ia^zygotene^, St.Ib^pachytene^, and St.Ib^diplotene^ stages, while PF-ii, PF-iii, and PF-iv were a more nuanced division of St.Ib^diplotene^ stages.

### 4.3. PF-ii to PF-iii Represents a Major Regulatory Transition

Ovarian development is a sophisticated, multifaceted biological process encompassing oocyte maturation, folliculogenesis, hormonal modulation, and spatiotemporal gene expression regulation [43,44]. During the transformation of zebrafish PFs to SFs, the oocytes begin to accumulate a large amount of yolk granules and other nutrients. Previous studies have suggested that this accumulation process may be one of the key rate-limiting steps in follicle development, and abnormal regulation may lead to follicle closure and ovarian function decline [45,46]. In this study, Transcriptomic, methylation, and WGCNA analyses collectively indicated that PF-ii to PF-iii represents a key developmental “switch,” during which follicles shift from a maintenance state toward anabolic activation. This transition coincides with the emergence of lampbrush chromosomes and increased vesicular activity, consistent with the onset of cortical alveoli formation and metabolic activation. Therefore, our data suggest that this process from PF-ii to PF-iii might be a crucial biological stage in the follicle development of zebrafish.

### 4.4. Coordinated Roles of Notch and mTOR in PF Progression

The Notch signaling pathway plays an important role in mammalian ovarian follicle development, inhibiting Notch signaling in cultured mouse follicles in vitro, leading to granulosa cell detachment and complete termination of follicle development [47,48,49]. The mTOR signaling pathway is key in individual growth and development, cell proliferation, and metabolism regulation [50]. In mice, the PI3K-Akt-mTOR signaling cascade regulates the resumption of oocyte meiosis, affecting maternal mRNA translation and early embryonic development, especially the activation of primordial follicles [51]. At 90 days post-fertilization (dpf), *esr1*-deficient female zebrafish exhibited overactivation of both the steroidogenic pathway and the mTOR signaling pathway, resulting in enhanced fertility and normal ovarian histology [52]. In our study, both transcriptomic profiling and functional perturbations support a role for these pathways in PF progression. Notch inhibition resulted in ovarian retardation and reduced mTOR activity, whereas amino acid supplementation (methionine + lysine) enhanced mTOR signaling and promoted follicle development.

Studies have shown that the nutritional components of feed can also affect ovarian development. After mTORC1 is activated by nutrients such as leucine, arginine, and methionine-derived methyl donor S-adenosylmethionine, it promotes the biosynthesis of proteins and lipids [53,54]. Based on these findings, we propose a working model in which Notch maintains granulosa–oocyte communication and PF identity during the PF-i to PF-ii stage. During the PF-ii to PF-iii transition, reprogramming of Notch activity may release inhibitory constraints and facilitate mTOR activation. Activated mTOR likely promotes protein synthesis, ribosome biogenesis, and endocrine responsiveness, enabling follicles to acquire vitellogenic competence. This model aligns with previous studies in mammals and offers a mechanistic basis for nutritional regulation of early follicle development.

### 4.5. Integration of Endocrine and Nutritional Regulation and Future Perspectives

Classical endocrine studies have established that pituitary-derived factors are essential for PF activation [55]. Our findings extend this model by showing that intrinsic follicular signaling—particularly the Notch–mTOR axis—functions as a regulatory interface that integrates endocrine inputs with environmental and nutritional cues. The observed enhancement of mTOR activity following amino acid supplementation indicates a nutrient-sensing mechanism that modulates PF developmental competence and may ultimately influence reproductive timing. Despite these insights, several limitations remain. Although methylation, transcriptional changes, and pathway activity showed consistent correlations, causal relationships have yet to be experimentally validated. Future work should incorporate cell-type-specific manipulation of Notch–mTOR components, assess downstream translational and endocrine responses, and extend comparative validation to additional aquaculture species to determine the broader applicability of this regulatory model.

## 5. Conclusions

In this study, we focused on the four PF subtypes (PF-i, PF-ii, PF-iii, and PF-iv) involved in zebrafish ovary development, describing their respective morphological characteristics and physiological biochemical features. Furthermore, through integrated multi-omics sequencing, we identified that the Notch/mTOR signaling pathway may be a central regulatory mechanism operating during critical developmental stages of PFs. These findings provide a robust theoretical foundation for enhancing reproductive efficiency in farmed fish populations and supporting precision breeding practices in aquaculture.

## Data Availability

Data will be made available on request. Transcriptome data have been uploaded to NCBI (https://www.ncbi.nlm.nih.gov/) (PRJNA1169999).

## Supplementary Materials

The following supporting information can be downloaded at: https://www.mdpi.com/article/10.3390/biology14121752/s1, Figure S1: lassification of primary follicles (PFs) in zebrafish; Figure S2: Zebrafish PF-i chromosome display; Figure S3: Observed and quantity statistics follicular cells of four subtypes of primary follicles (PFs) in zebrafish; Figure S4: Transcriptome data supplement; Figure S5: Distribution in the genome of differentially methylated sites in PF-i to PF-ii, PF-ii to PF-iii, and PF-iii to PF-iv stage. Figure S6: Correlation between expression levels and CG methylation levels of differential genes in PF-i to PF-ii, PF-ii to PF-iii, and PF-iii to PF-iv stage; Table S1: Primer sequences used for polymerase chain reaction; Table S2: Measurement size of the follicles from Percoll density gradients; Table S3: Base quality analysis of filtered data; Table S4: Refer to the statistical table of genome comparison analysis results. The original Western blot images supporting these results are shown in the Original Images.
