# Supplementary material for: Primary Follicle Paces Fish Ovarian Maturation Developmental Progression via the Enhancement of Notch and mTOR
Source: Biology (Basel). 2025 Dec 6;14(12):1752. doi: 10.3390/biology14121752 (PMC12730785; doi:10.3390/biology14121752)
Supplement: Supplementary file 1 [file biology-14-01752-s001.zip › Supplementary Material/Supplementary Material (Figures S1-S6).pdf]

Figure Legends

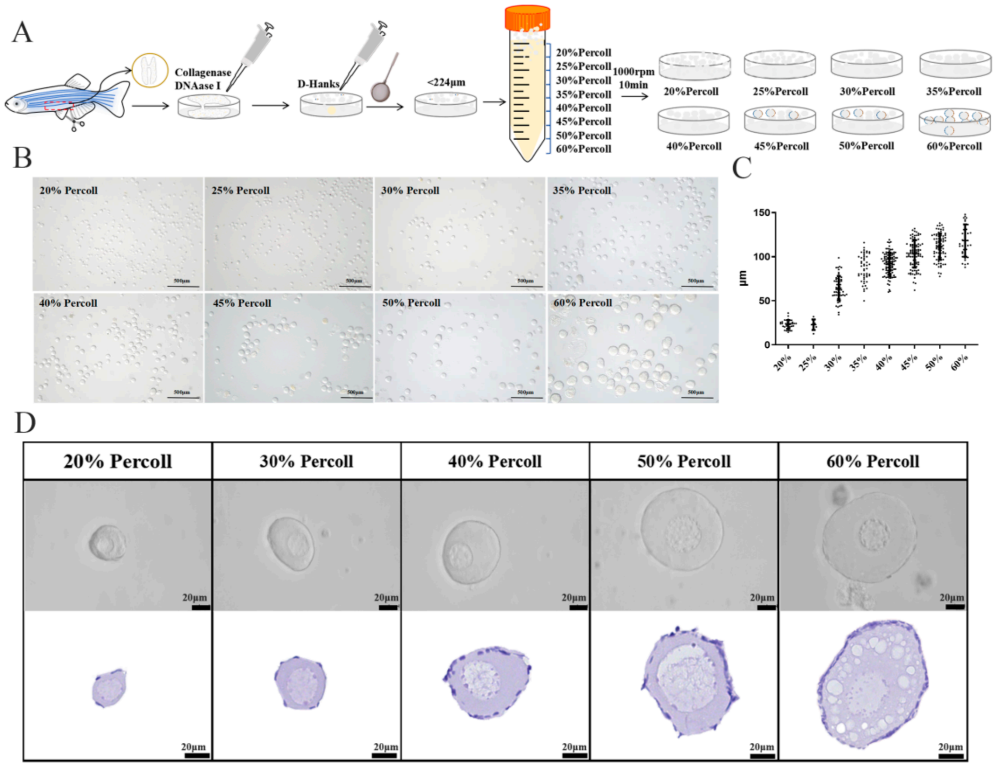

Figure S1. Classification of primary follicles (PFs) in zebrafish.

(A) Flow chart of separation and purification of zebrafish follicles using NaCl-Percoll density gradient centrifugation. (B) Observation of the follicle clusters from 20%, 25%, 30%, 35%, 40%, 45%, 50%, and 60% Percoll under a phase contrast microscope. (C) The diameter length statistics follicle clusters from 20%, 25%, 30%, 35%, 40%, 45%, 50%, and 60% Percoll. (D) Observation of invitro follicles (previous row) and their histological sections (next row)from 20%, 30%, 40%, 50%, and 60% Percoll. Scale bar, 20 μm. The follicles histological stained with hematoxylin and eosin.

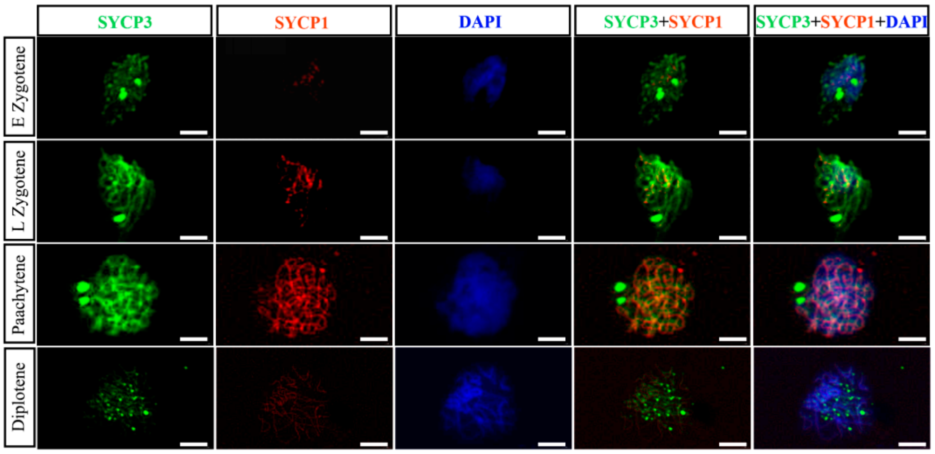

Figure S2. Zebrafish PF-i chromosome display

"E" indicating "early"; "L" indicating "late"; Green: SYCP3; Red: SYCP1; Blue: DAPI; Scale: 5μm.

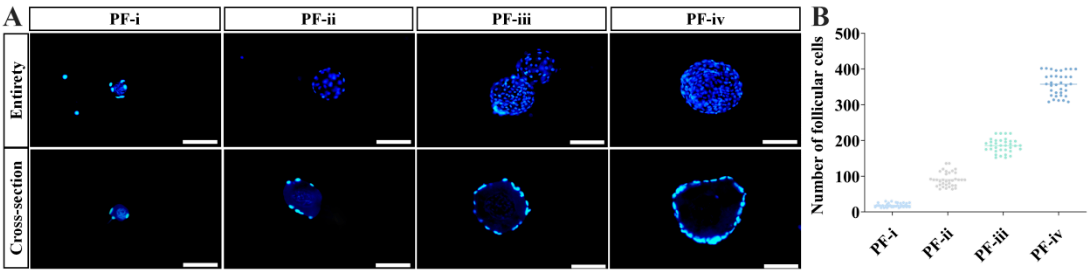

Figure S3. Observed and quantity statistics follicular cells of four subtypes of primary follicles (PFs) in zebrafish.

(A) Follicular cells were observed in invitro follicles (previous row) and their histological sections (next row) by DAPI staining (blue). Scale bar, 50 μm. (B) The number of follicular cells in four subtypes of PFs.

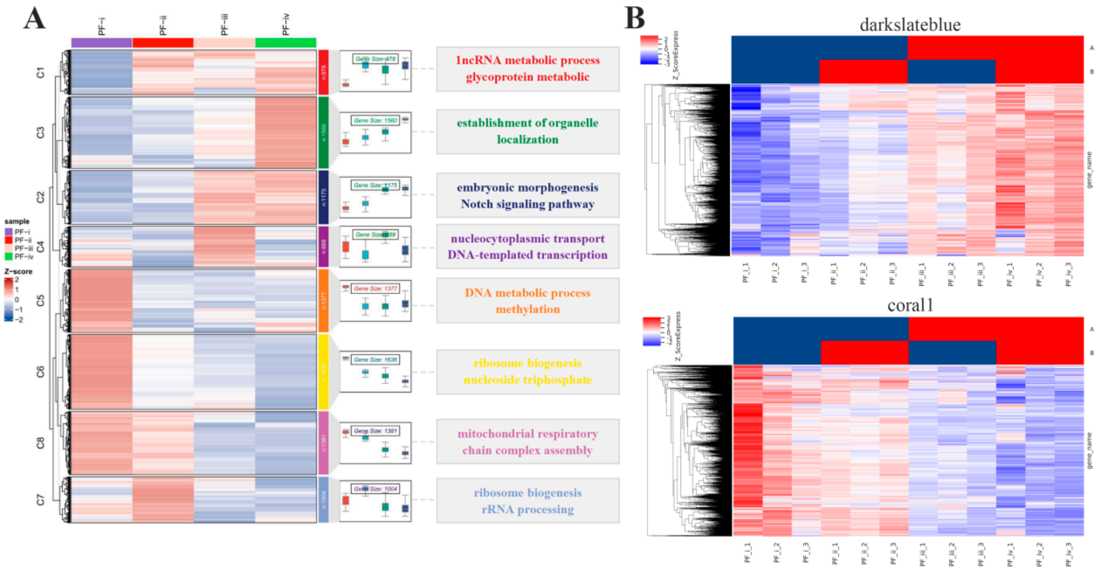

Figure S4. Transcriptome data supplement.

(A) The getClusters function analyzed heat maps of gene expression patterns in 8 clusters for several GO enrichment analyses. (B) Gene expression heat maps of medium darkslateblue and coral modules analyzed by WGCNA.

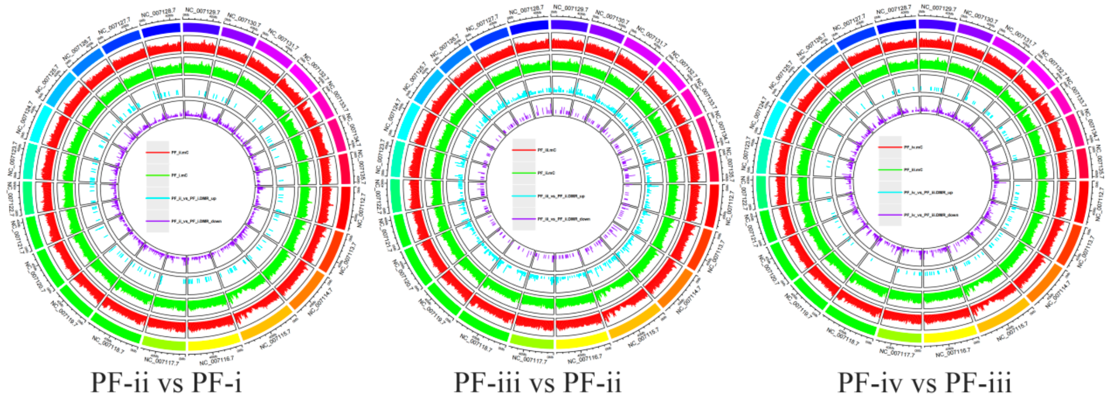

Figure S5. **Distribution in the genome of differentially methylated sites in PF-i to PF-ii, PF-ii to PF-iii, and PF-iii to PF-iv stage.**

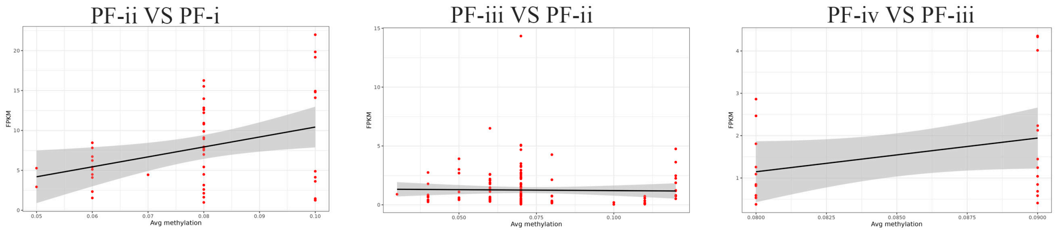

Figure S6. **Correlation between expression levels and CG methylation levels of differential genes in PF-i to PF-ii, PF-ii to PF-iii, and PF-iii to PF-iv stage.**
