# Supplementary material for: Primary Follicle Paces Fish Ovarian Maturation Developmental Progression via the Enhancement of Notch and mTOR
Source: Biology (Basel). 2025 Dec 6;14(12):1752. doi: 10.3390/biology14121752 (PMC12730785; doi:10.3390/biology14121752)
Supplement: Supplementary file 1 [file biology-14-01752-s001.zip › Supplementary Material/Supplementary Material (Tables S1-S4).pdf]

Table S1. Primer sequences used for polymerase chain reaction.

Genes	Forward primers	Reverse primers
notch2	GCCGGCAAACAACAGAAACCAC	TCCATTCAGGTGGGCACTGGC
notch3	CCGGTGACCACACCTACGAATG	CACTCAGGAGGGCACTGGCAA
akt1	GCTGTGCTGCTGTTGTCTTC	TGTGGAGGCTGCTGTGATTA
akt2	AGCCATCTTGAGGATCTTGGA	TCTTGGTCCGTTTCTGCTTCT
akt3a	CGGCTCTACTCTGAGAACCA	CCTTCTGCTTCTTCTTCCCA
akt3b	GCTTCTCTGCTGAGTCTCCA	CTTCTCTCCAGGTCTTCTCCA
mtor	CCGGATTTCTGAGCTGACTT	CGGTCAAGGTGTCAGAGCAT
s6k1	CAGGAGCAGGTGAAGGAGAA	CTGCTCCTTCTCCTTCTCCA
hey1	TTGATGCTCACGCTCTGGCCA	AGCTTCTCTCTGAGAGGCGTAGC
hey2	GGCCATGGACTTCTTGAGCATCGG	CTATGGATGTGGTCATGGCGGC
hes	CCCACTGGTCGGCAAAGATGC	GCTGCAAGCAGGCTTTATAGCCC
β-actin	AGCCGAAAGAGAAATTGTCC	GAAACGCTCATTGCCGAT

Table S2. Measurement size of the follicles from Percoll density gradients.

Percoll (%)	Mean ± standard deviation (μm)	(min, mix) (μm)
20%	22.945±5.955	(12.649，29.120)
25%	23.265±4.946	（14.422，36.221）
30%	64.356±13.840	（34.408，82.817）
35%	86.909±15.037	（60.120，110.345）
40%	88.948±14.663	（76.942，116.825）
45%	103.343±15.617	（80.895，125.539）
50%	111.487±14.809	（91.214，128.063）
60%	118.358±18.311	（98.061，145.986）

Table S3. Base quality analysis of filtered data

Sample	RawReads	RawBases	CleanReads	CleanBases	CleanRatio	Q20	Q30	GC
PF-i-1	284887468	42733120200	227009626	31349358032	79.68%	98.97%	96.89%	19.68%
PF-i-2	287015160	43052274000	243666418	33547261828	84.90%	98.67%	95.68%	19.35%
PF-i-3	291991474	43798721100	245103218	33728458844	83.94%	98.63%	95.53%	19.44%
PF-ii-1	283968138	42595220700	237922412	32734326628	83.78%	98.67%	95.73%	19.80%
PF-ii-2	297179522	44576928300	251220690	34598458202	84.53%	98.72%	95.86%	19.88%
PF-ii-3	304029356	45604403400	254284156	35004191876	83.64%	98.66%	95.68%	19.91%
PF-iii-1	294700450	44205067500	246564420	33964865888	83.67%	98.69%	95.80%	19.52%
PF-iii-2	298858988	44828848200	246378358	33948412680	82.44%	98.74%	95.95%	19.45%
PF-iii-3	306631856	45994778400	257490638	35473857330	83.97%	98.73%	95.89%	19.71%
PF-iv-1	303294608	45494191200	255228230	35139192710	84.15%	98.71%	95.82%	19.83%
PF-iv-2	297322498	44598374700	247977208	34135243516	83.40%	98.71%	95.84%	20.02%
PF-iv-3	304958516	45743777400	256915894	35367320860	84.25%	98.67%	95.72%	20.00%

Table S4. Refer to the statistical table of genome comparison analysis results

Sample	TotalReads	MappedReads	MapRate	UniqReads	DupReads	EffectReads	EffectRate	ConversionRate
PF-i-1	227009626	154216250	67.93%	139441358	24811348	114629944	50.50%	99.64%
PF-i-2	243666418	161849914	66.42%	147104306	27290736	119813508	49.17%	99.60%
PF-i-3	245103218	164237444	67.01%	148755104	29383568	119371454	48.70%	99.59%
PF-ii-1	237922412	160026948	67.26%	144306730	26801076	117505548	49.39%	99.61%
PF-ii-2	251220690	169382228	67.42%	152639904	25362120	127277642	50.66%	99.60%
PF-ii-3	254284156	171591492	67.48%	154298806	28103180	126195442	49.63%	99.60%
PF-iii-1	246564420	166713144	67.61%	150664938	23097842	127566974	51.74%	99.59%
PF-iii-2	246378358	166128932	67.43%	150620286	27167516	123452644	50.11%	99.60%
PF-iii-3	257490638	177811538	69.06%	160884946	28142054	132742790	51.55%	99.60%
PF-iv-1	255228230	173965532	68.16%	156565560	26973552	129591634	50.77%	99.59%
PF-iv-2	247977208	170076546	68.59%	152630886	25888148	126742464	51.11%	99.58%
PF-iv-3	256915894	176858456	68.84%	158788916	27474086	131314614	51.11%	99.57%
