# Supplementary figures and images for: Primary Follicle Paces Fish Ovarian Maturation Developmental Progression via the Enhancement of Notch and mTOR
Source: Biology (Basel). 2025 Dec 6;14(12):1752. doi: 10.3390/biology14121752 (PMC12730785; doi:10.3390/biology14121752)

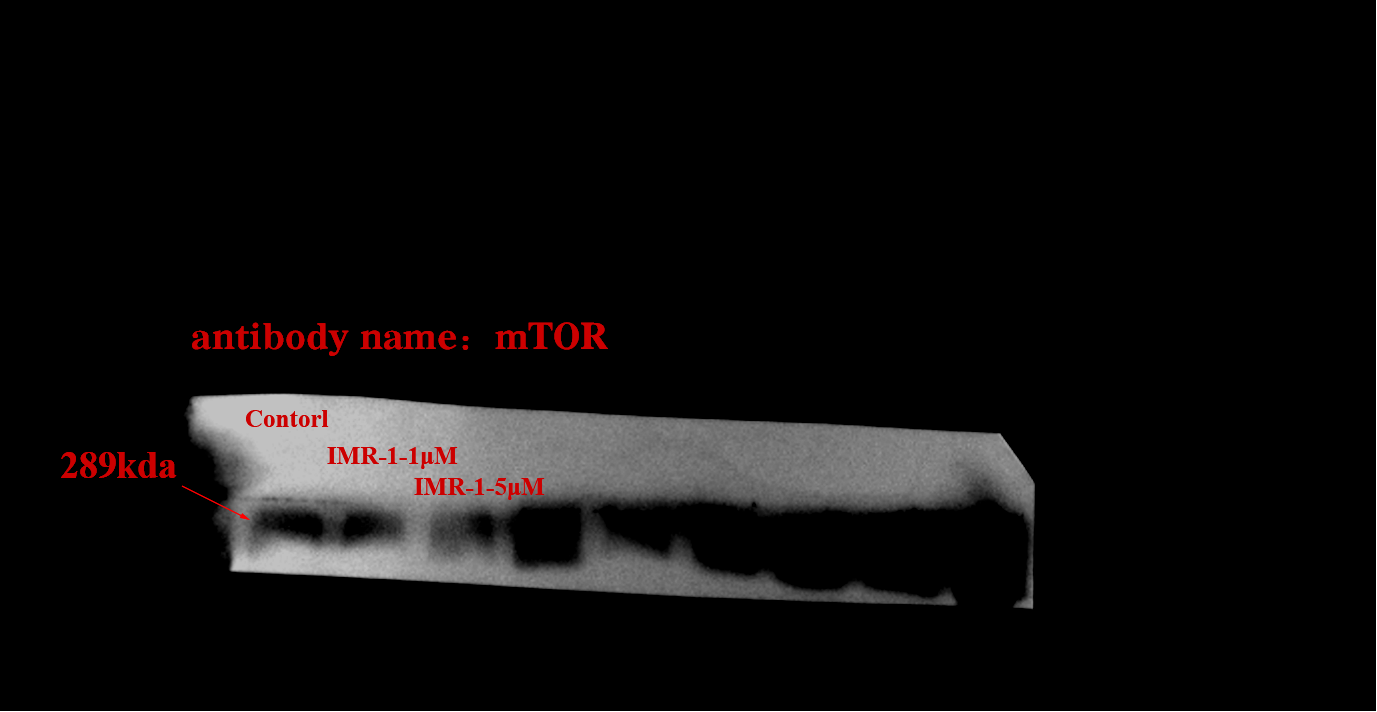

Supplement: Supplementary file 1 [file biology-14-01752-s001.zip › Original Images/F-Mtor.tif]

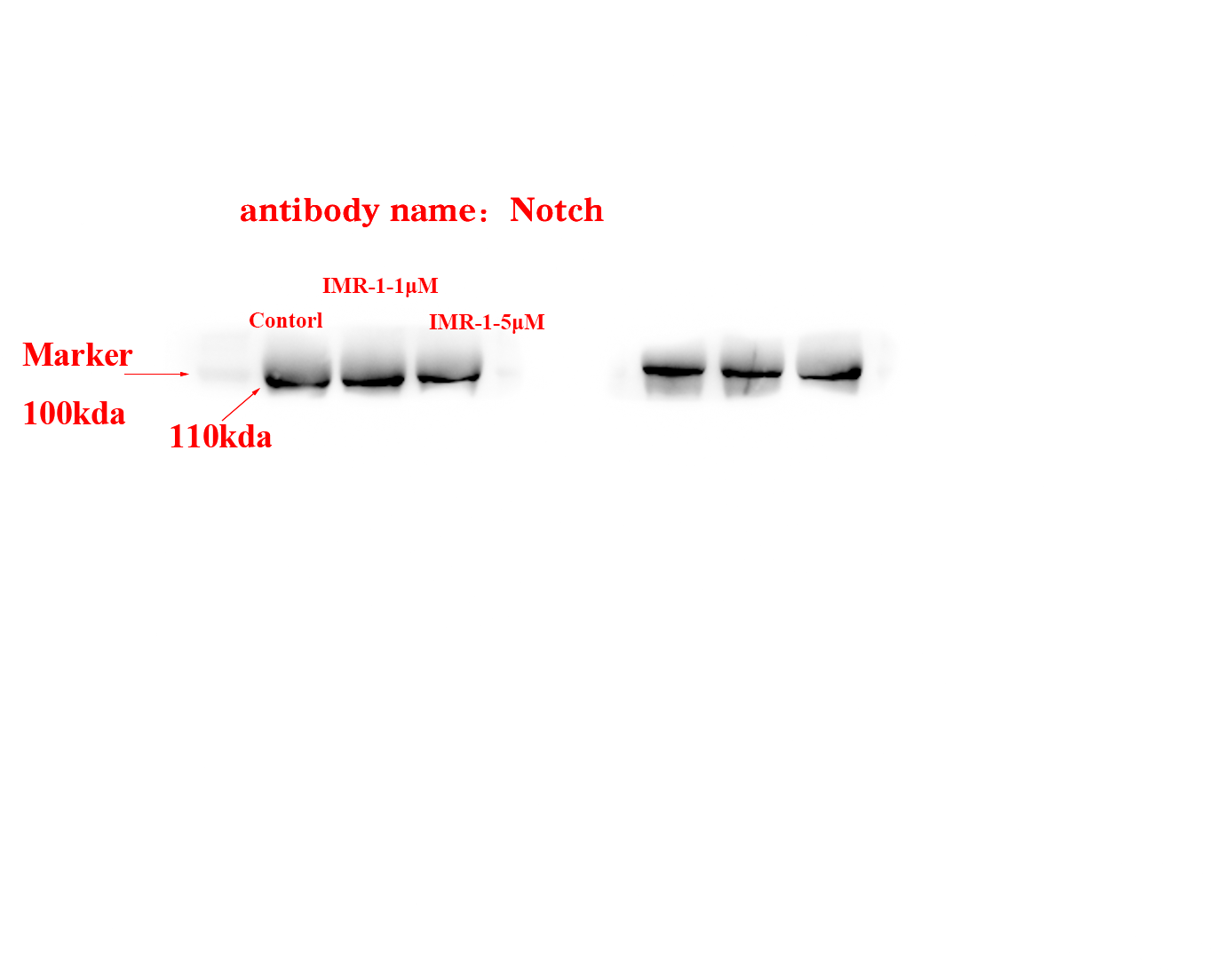

Supplement: Supplementary file 1 [file biology-14-01752-s001.zip › Original Images/F-notch.tif]

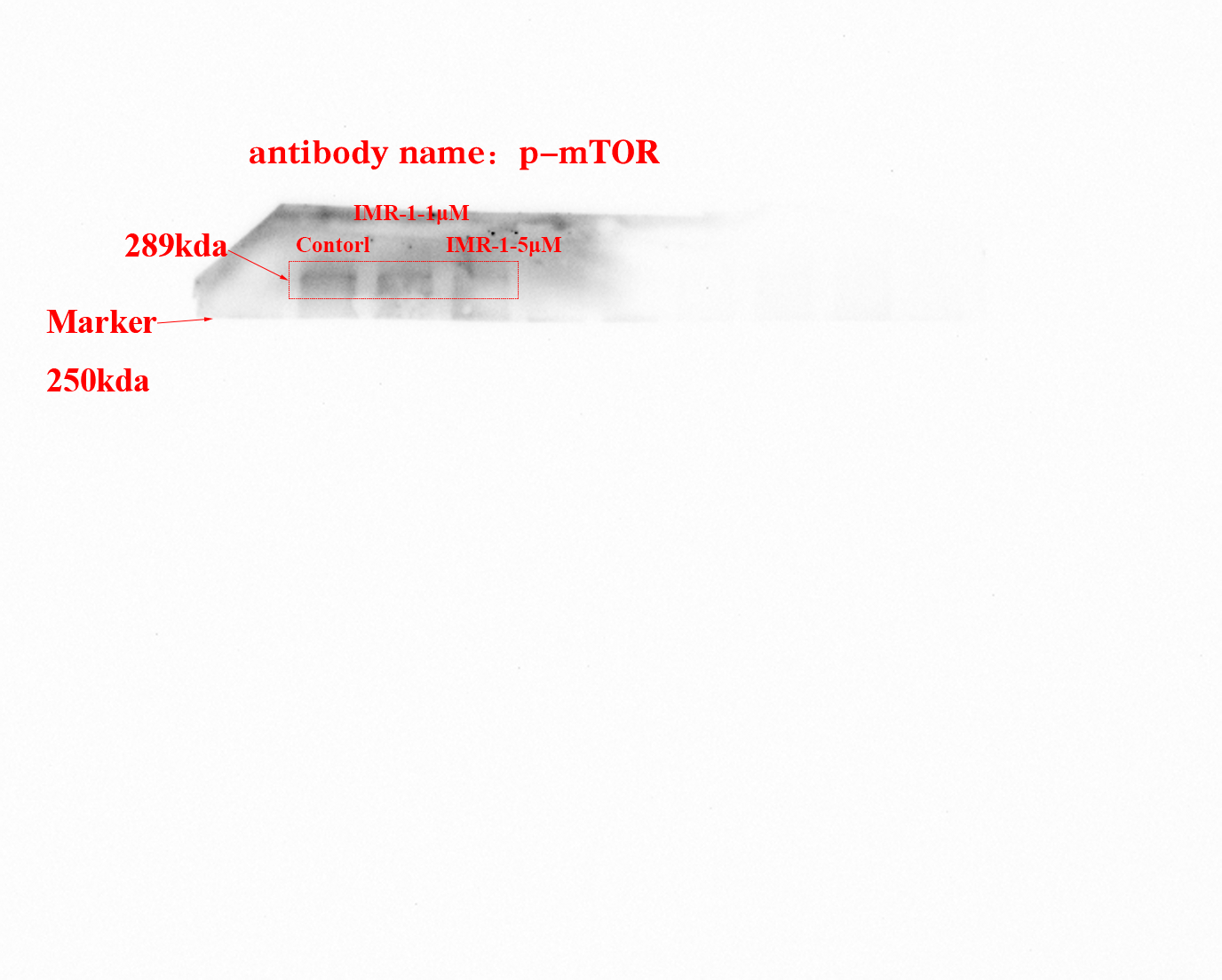

Supplement: Supplementary file 1 [file biology-14-01752-s001.zip › Original Images/F-p-Mtor.tif]

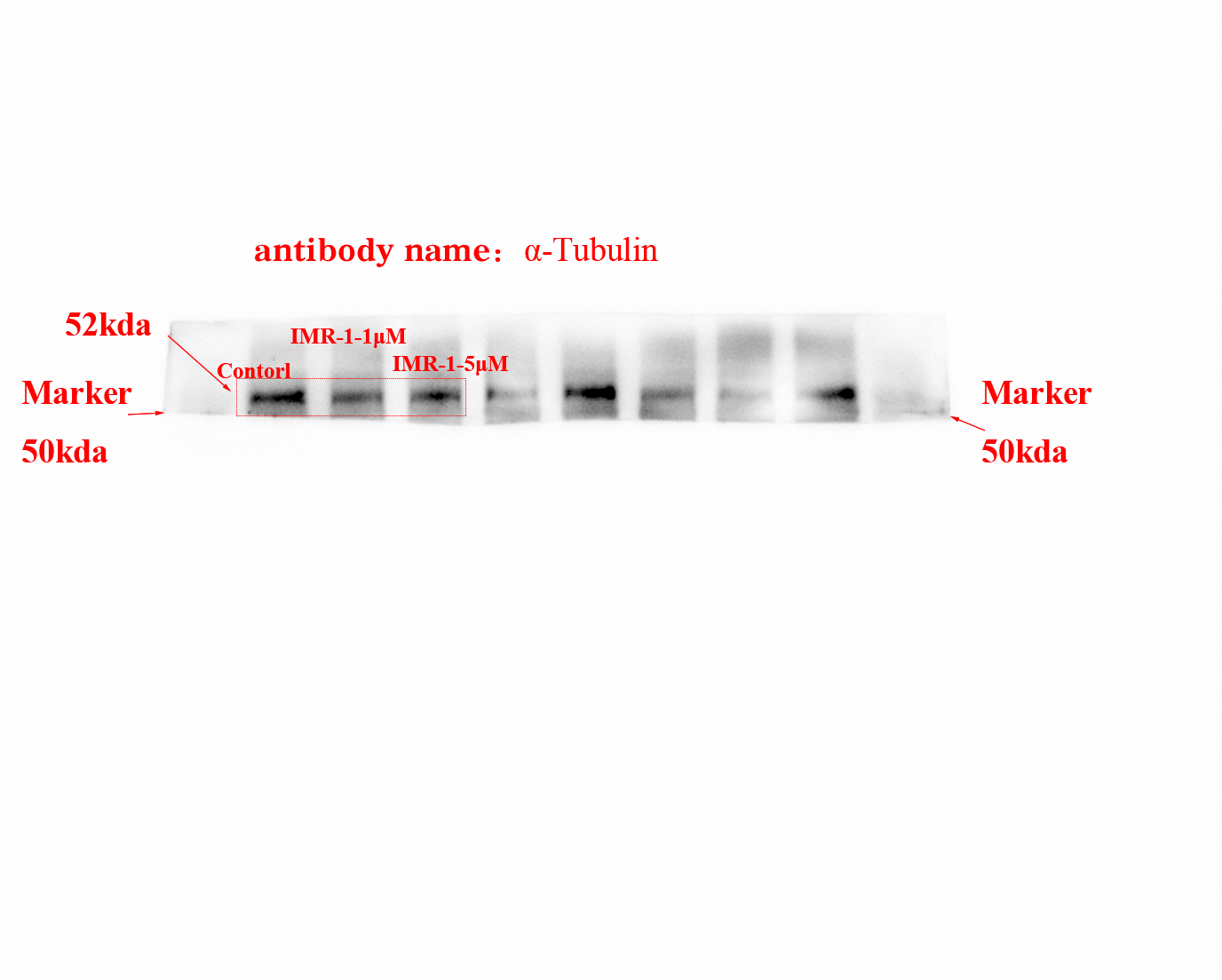

Supplement: Supplementary file 1 [file biology-14-01752-s001.zip › Original Images/F-tubulin.tif]

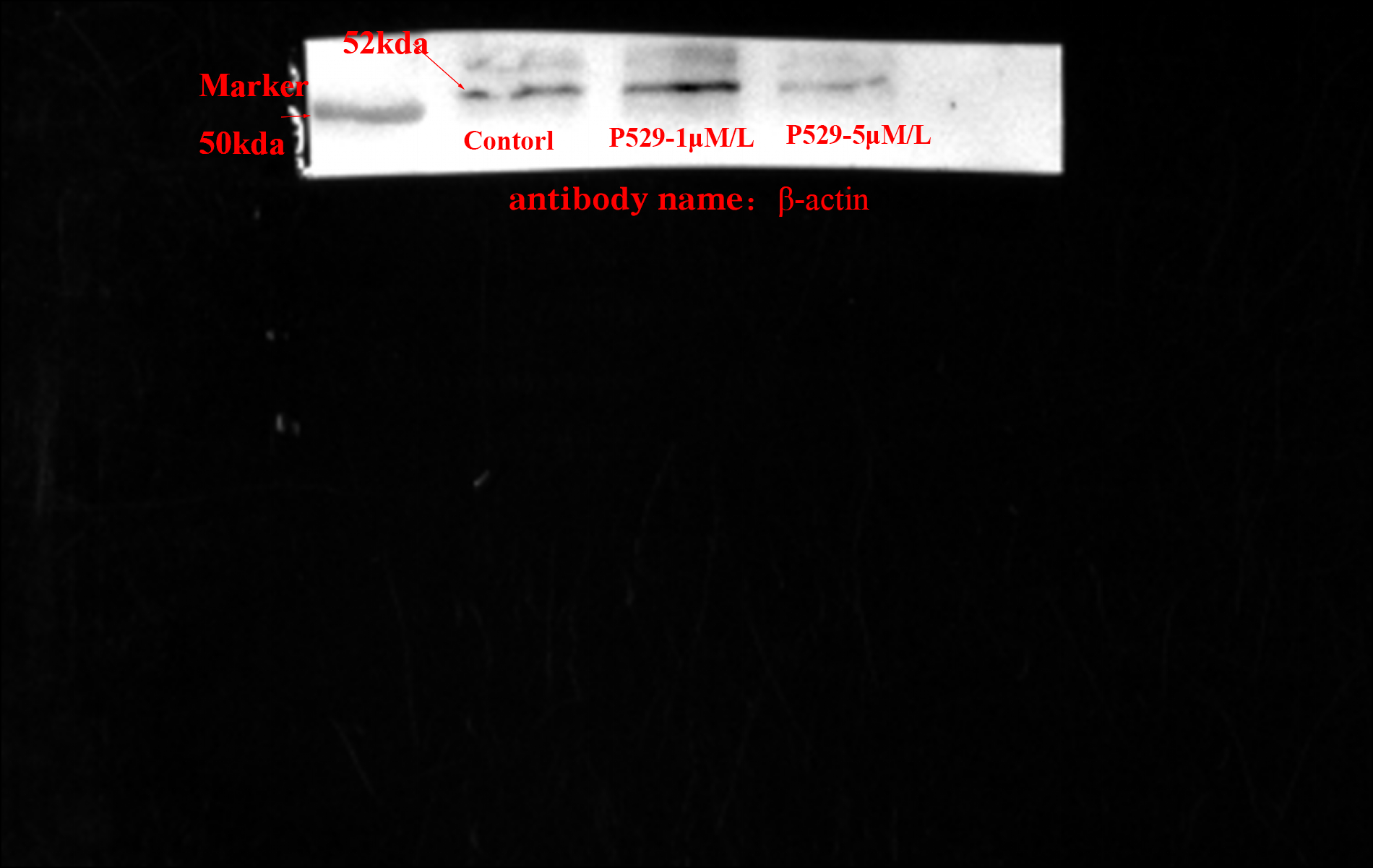

Supplement: Supplementary file 1 [file biology-14-01752-s001.zip › Original Images/I-Actin.tif]

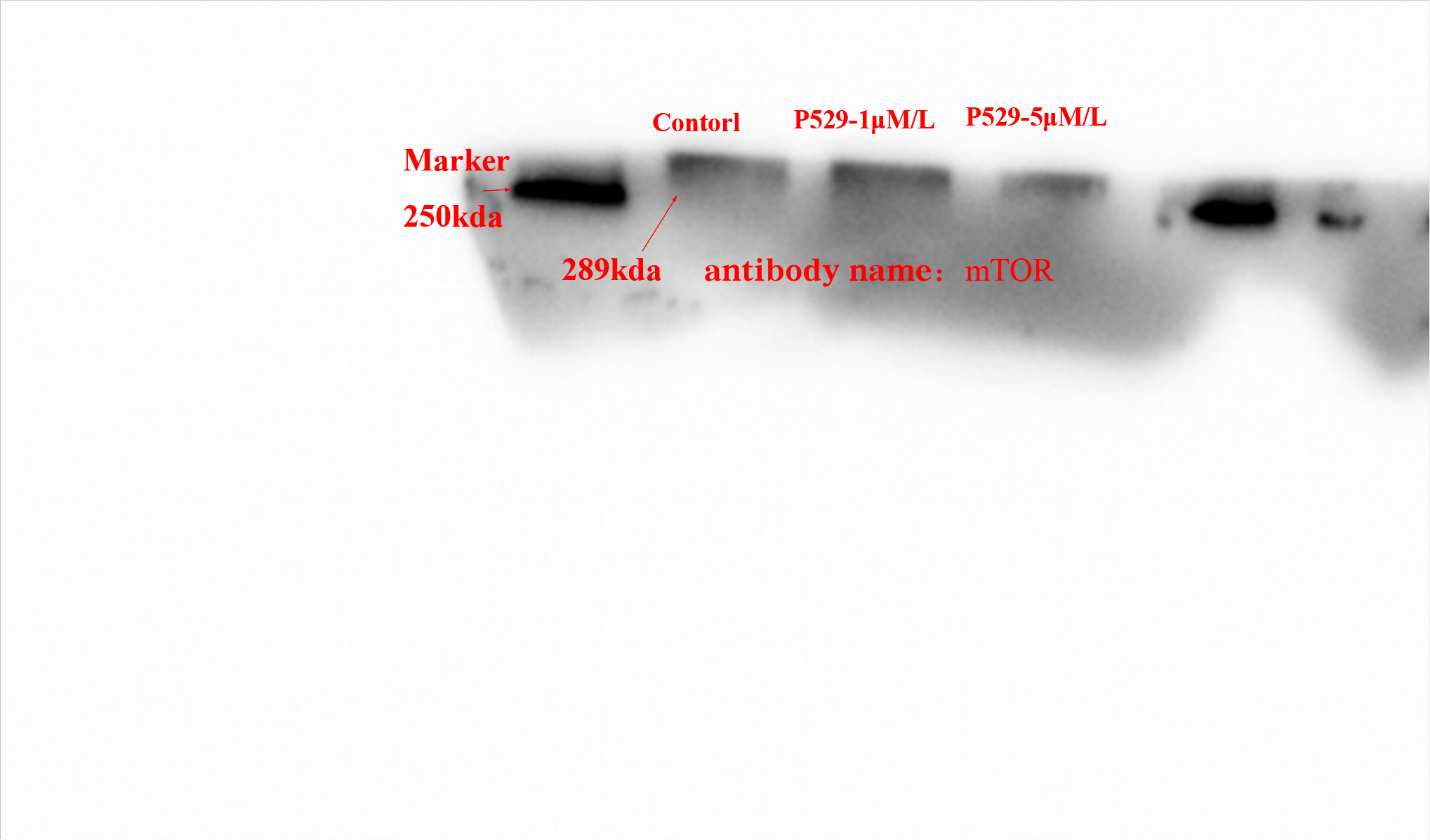

Supplement: Supplementary file 1 [file biology-14-01752-s001.zip › Original Images/I-mTOR.tif]

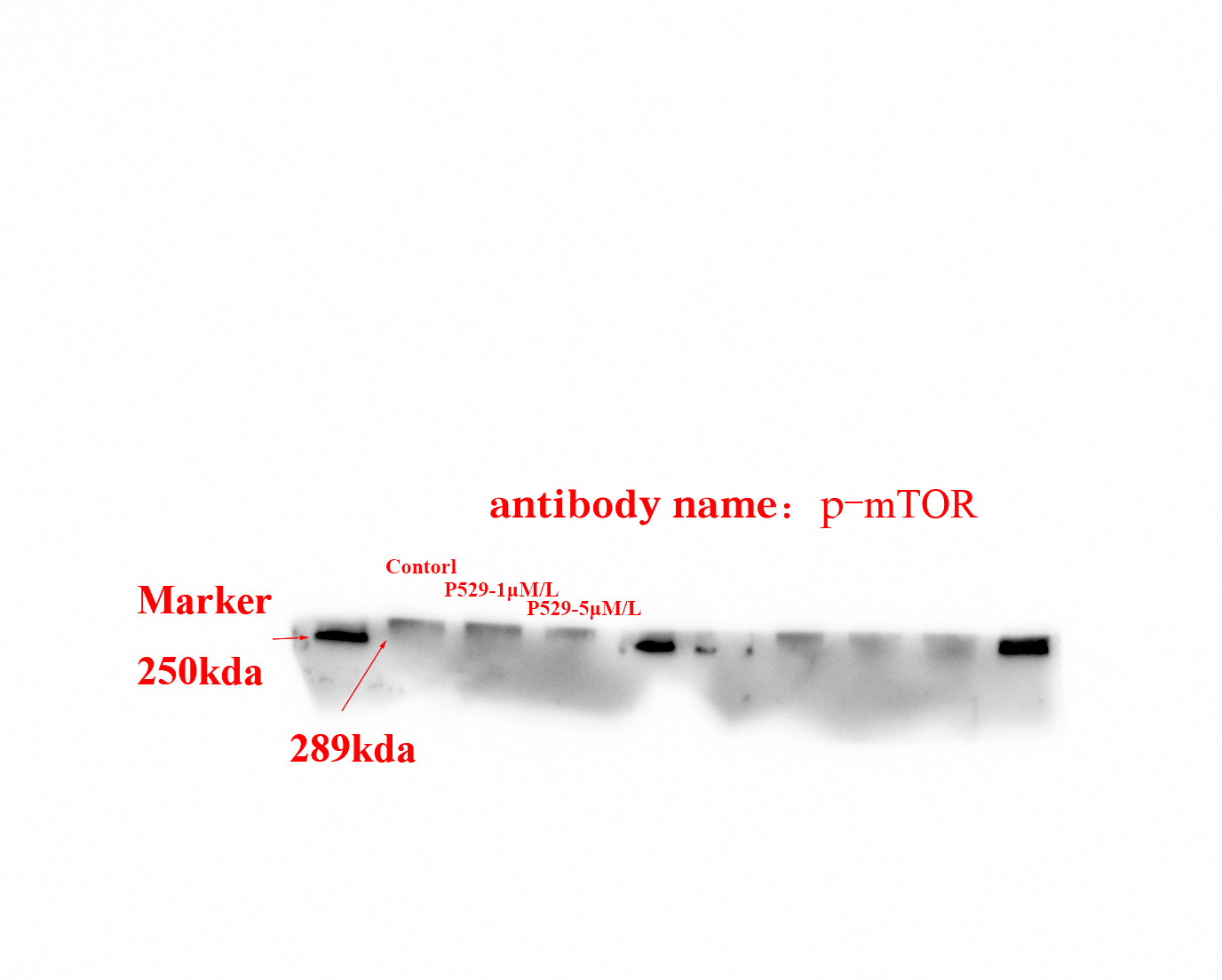

Supplement: Supplementary file 1 [file biology-14-01752-s001.zip › Original Images/I-p-mTOR-10-2.tif]

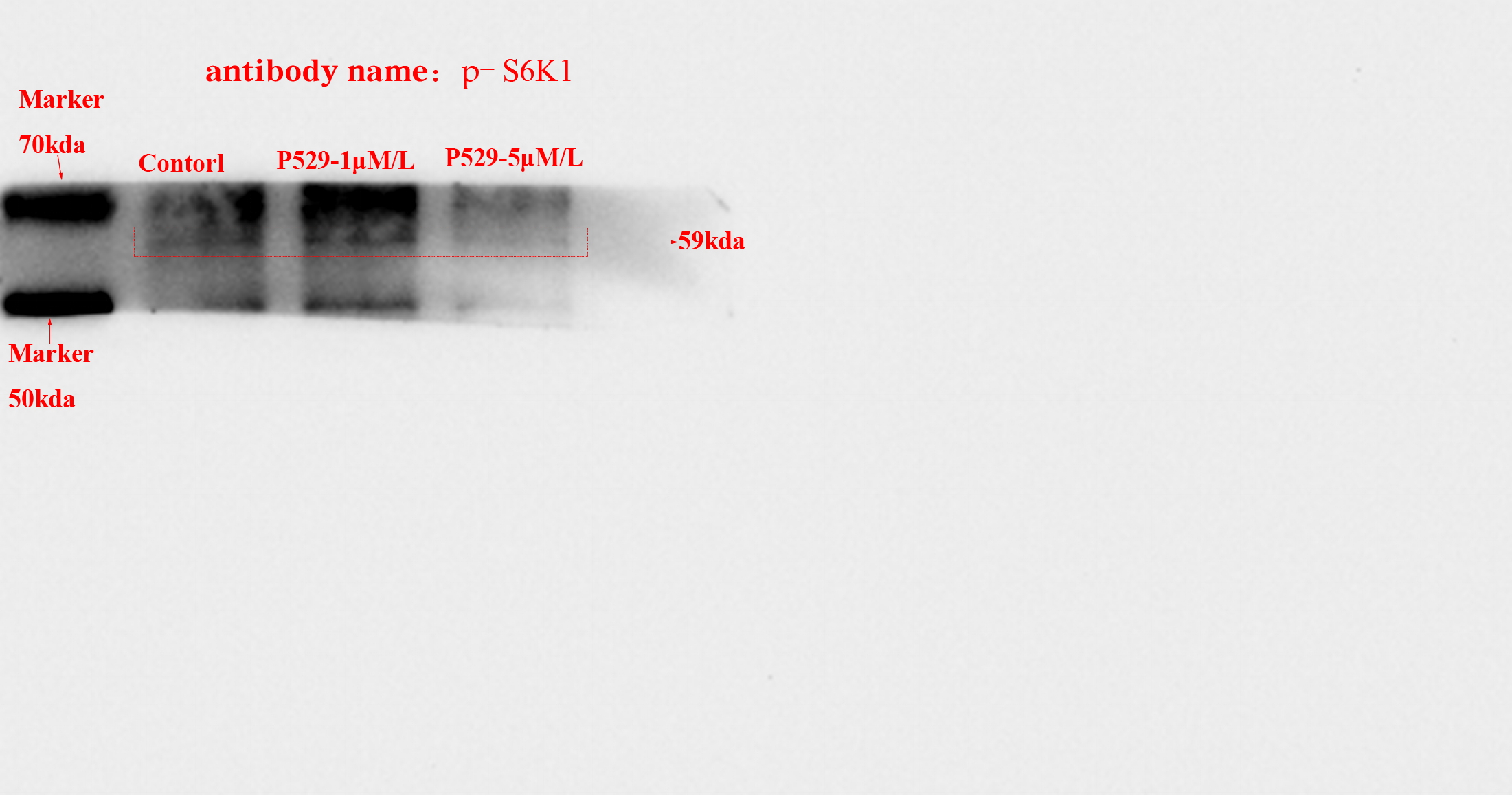

Supplement: Supplementary file 1 [file biology-14-01752-s001.zip › Original Images/I-p-S6K1.tif]

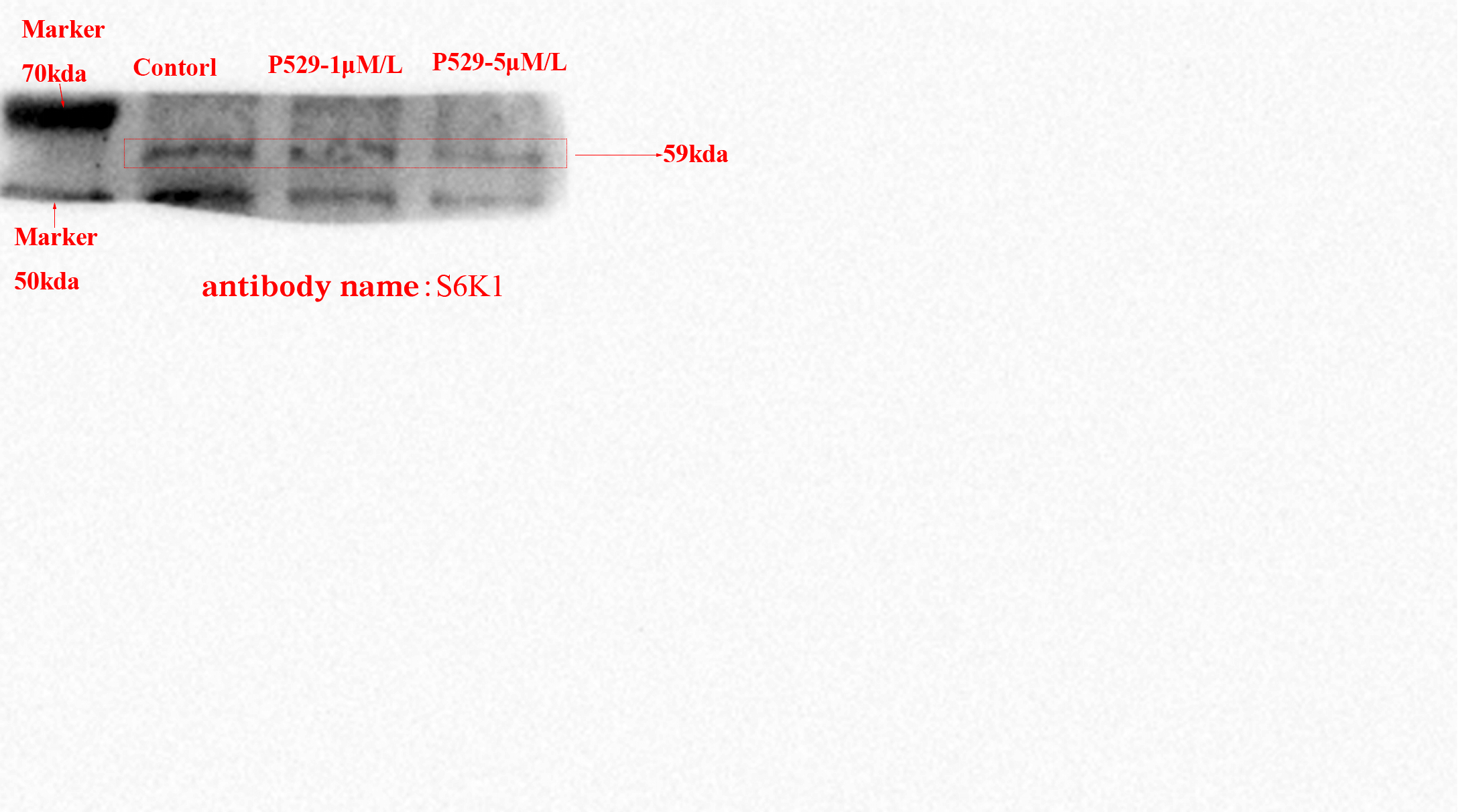

Supplement: Supplementary file 1 [file biology-14-01752-s001.zip › Original Images/I-S6K1.tif]

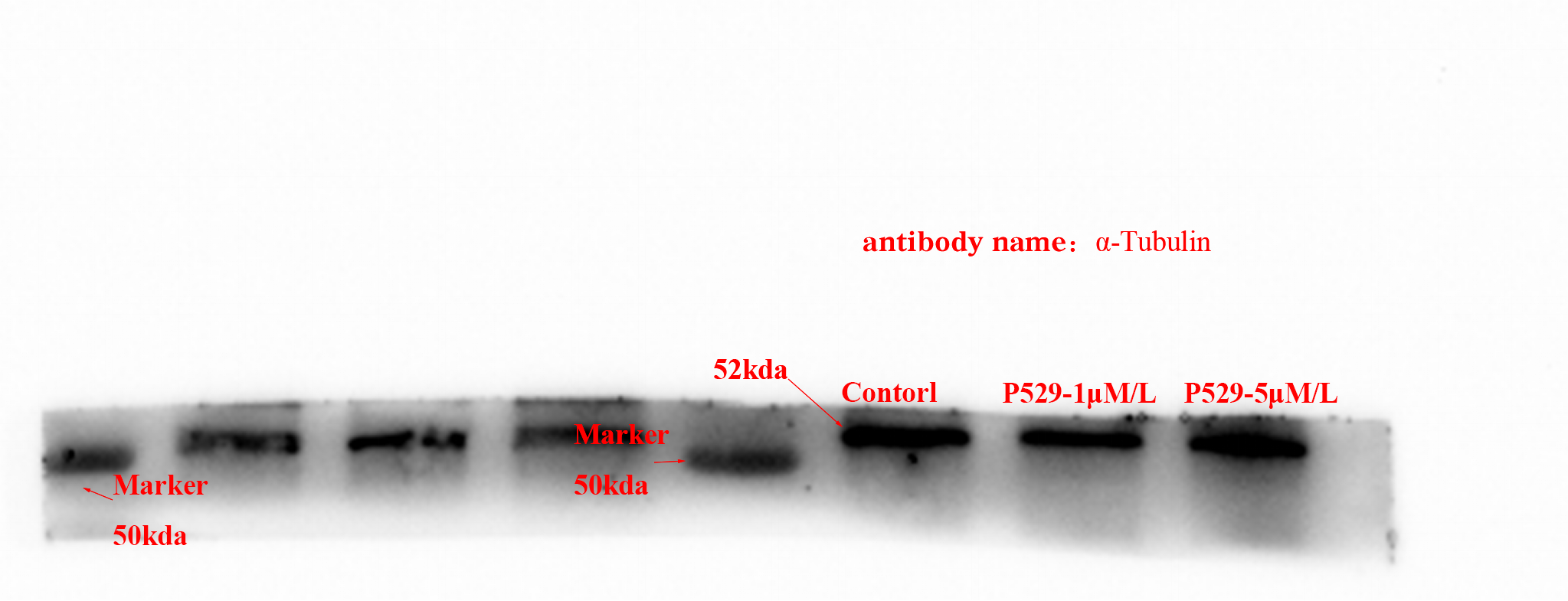

Supplement: Supplementary file 1 [file biology-14-01752-s001.zip › Original Images/I-Tubulin.tif]

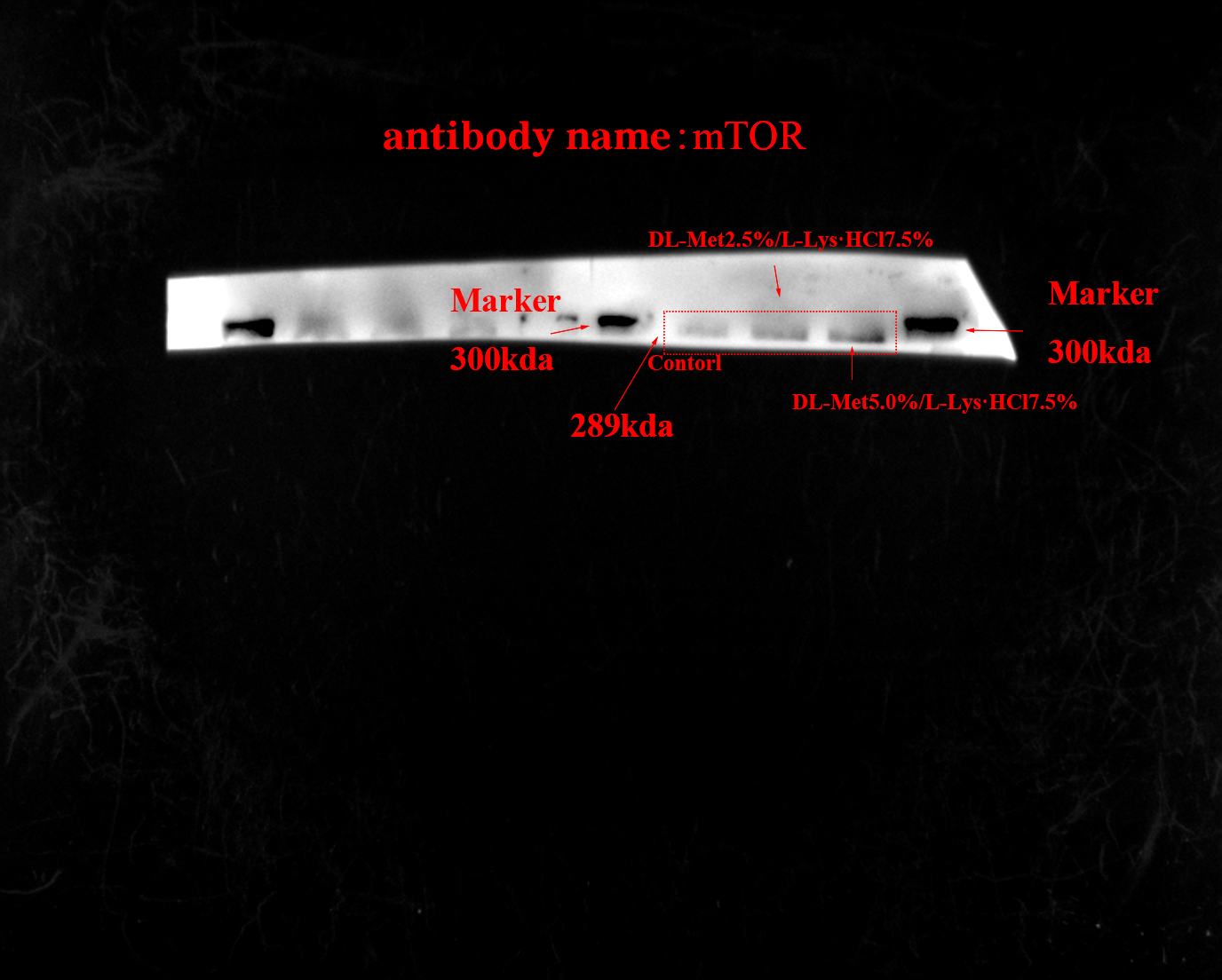

Supplement: Supplementary file 1 [file biology-14-01752-s001.zip › Original Images/L-mTOR.tif]

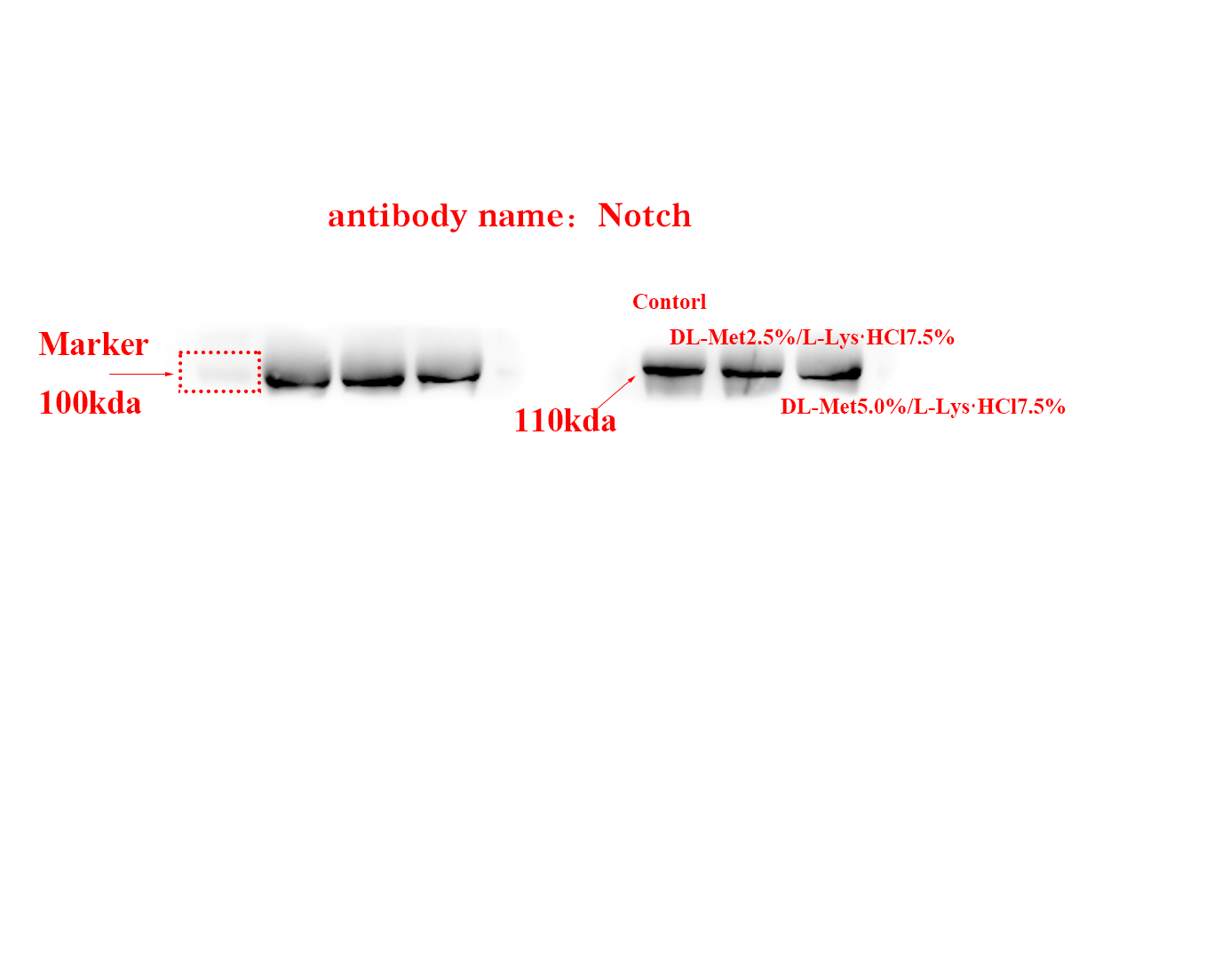

Supplement: Supplementary file 1 [file biology-14-01752-s001.zip › Original Images/L-notch.tif]

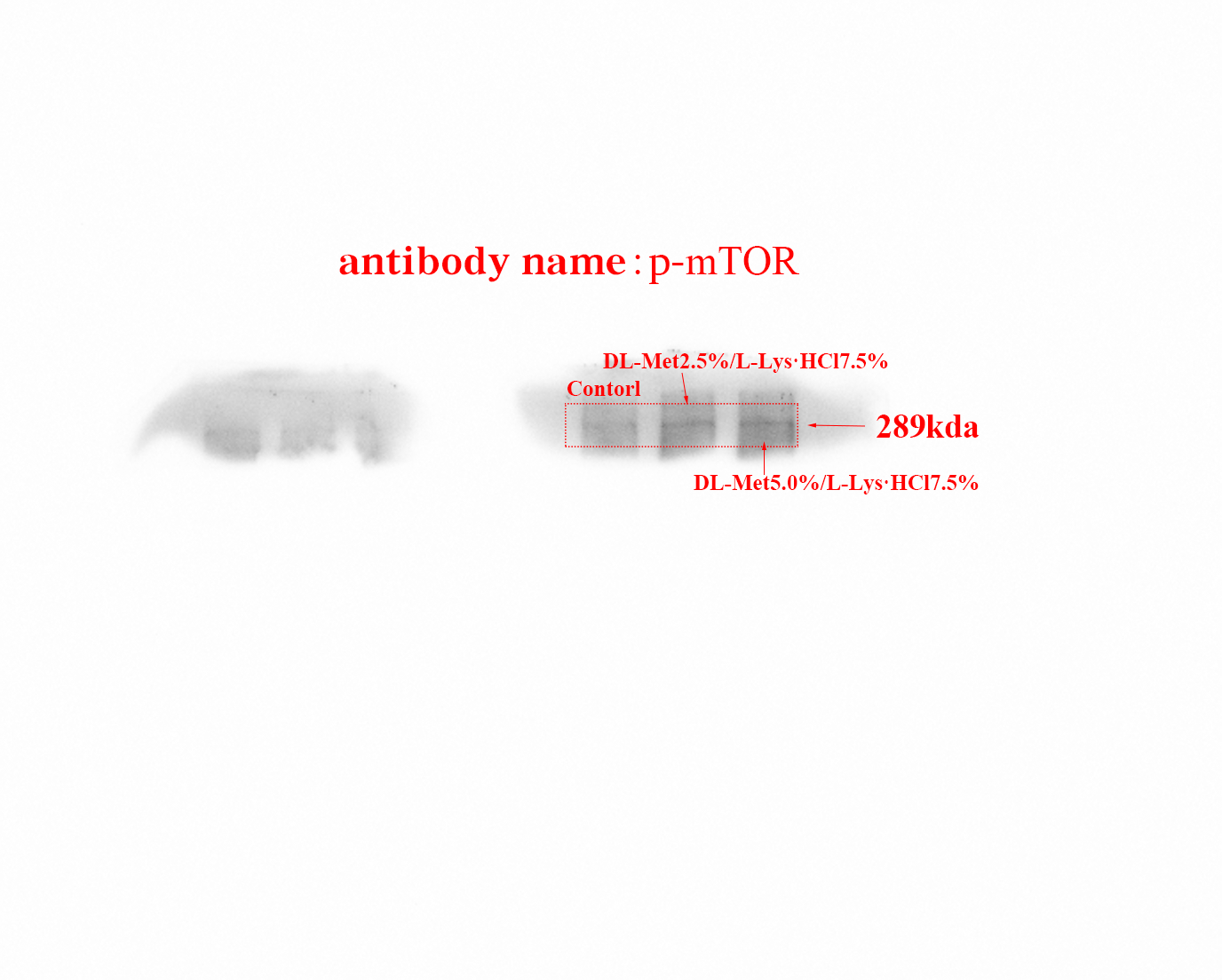

Supplement: Supplementary file 1 [file biology-14-01752-s001.zip › Original Images/L-p-mTOR-1.tif]

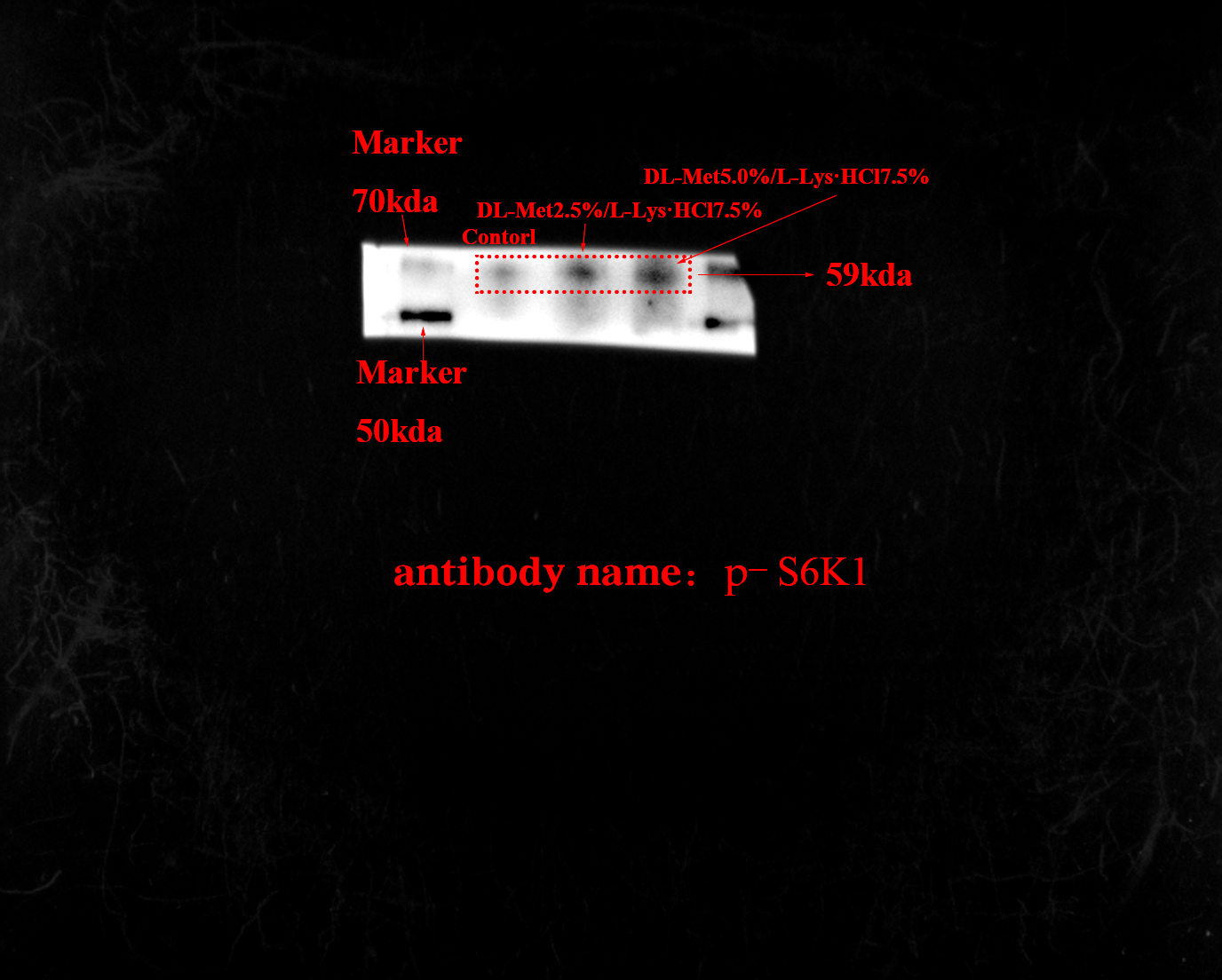

Supplement: Supplementary file 1 [file biology-14-01752-s001.zip › Original Images/L-p-s6k1.tif]

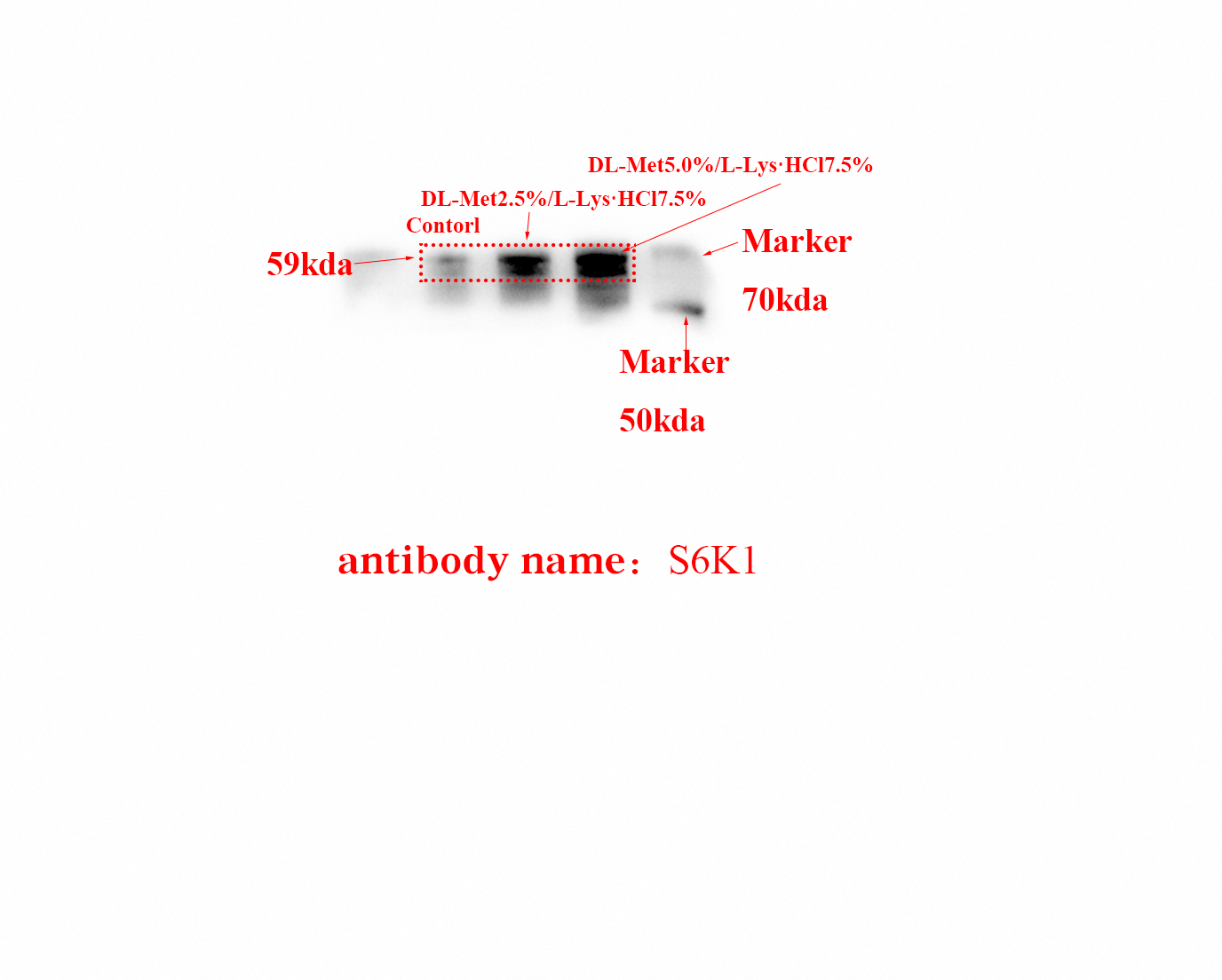

Supplement: Supplementary file 1 [file biology-14-01752-s001.zip › Original Images/L-s6k1.tif]

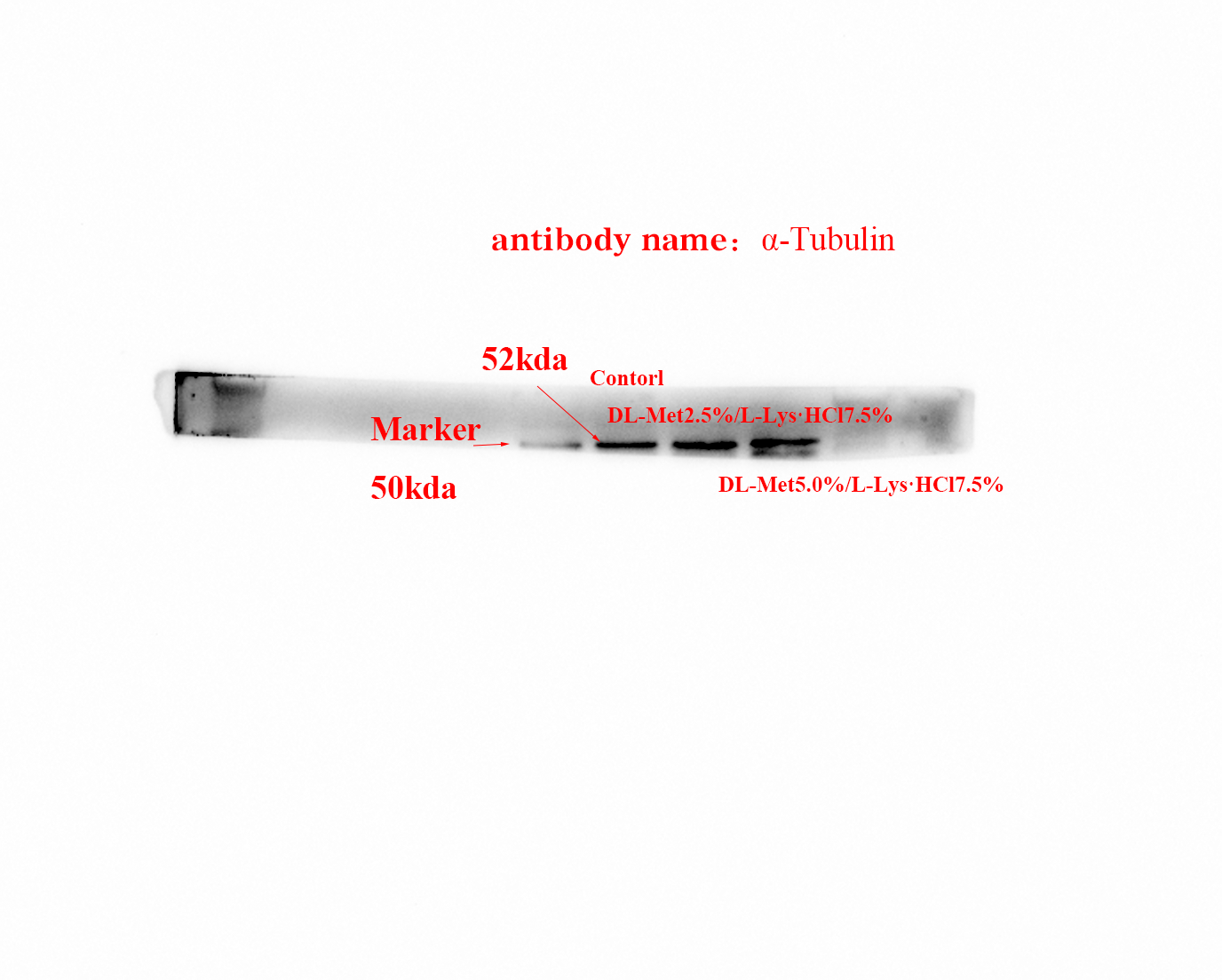

Supplement: Supplementary file 1 [file biology-14-01752-s001.zip › Original Images/L-tubulin.tif]

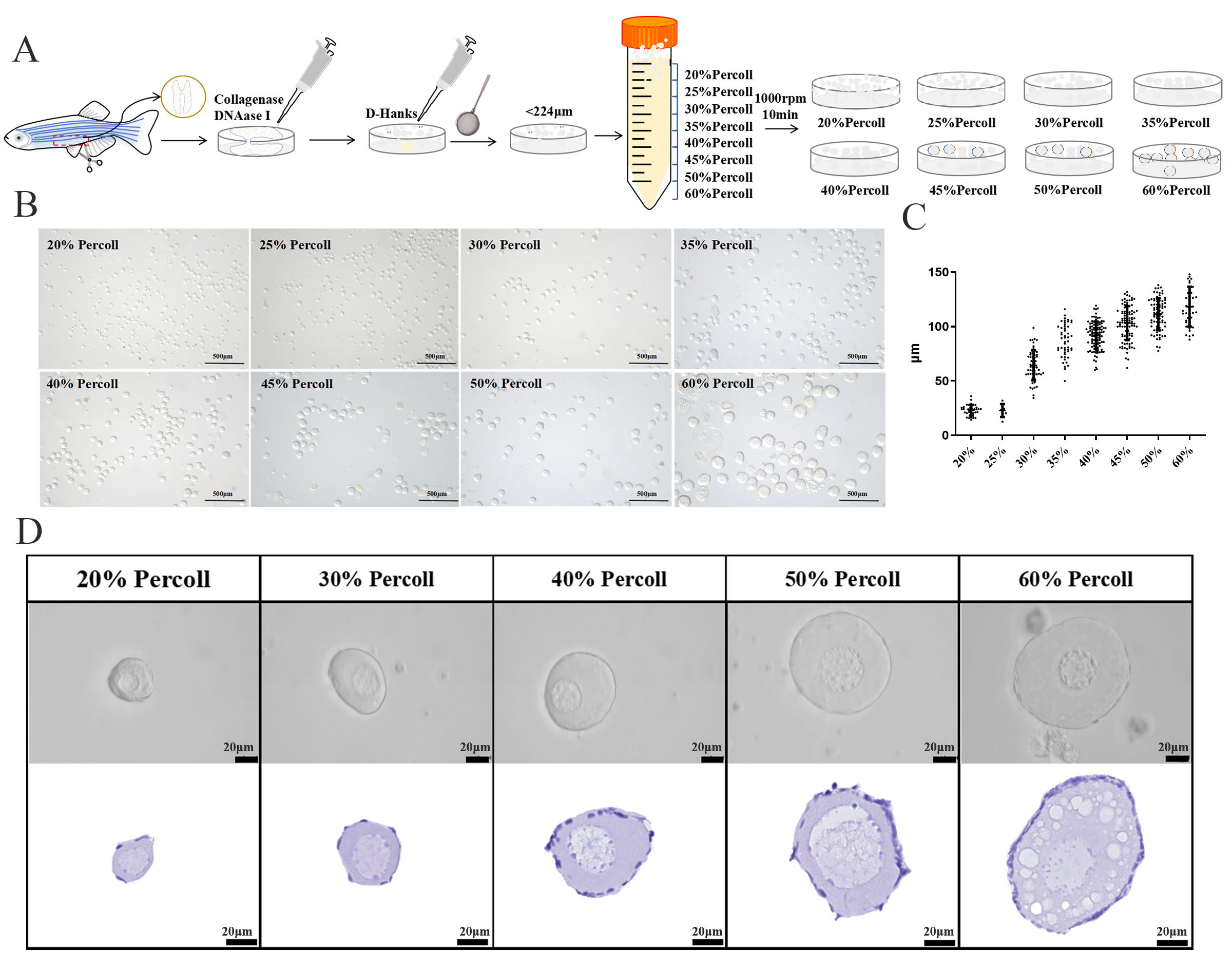

Supplement: Supplementary file 1 [file biology-14-01752-s001.zip › Supplementary Material/Fig. S1.tif]

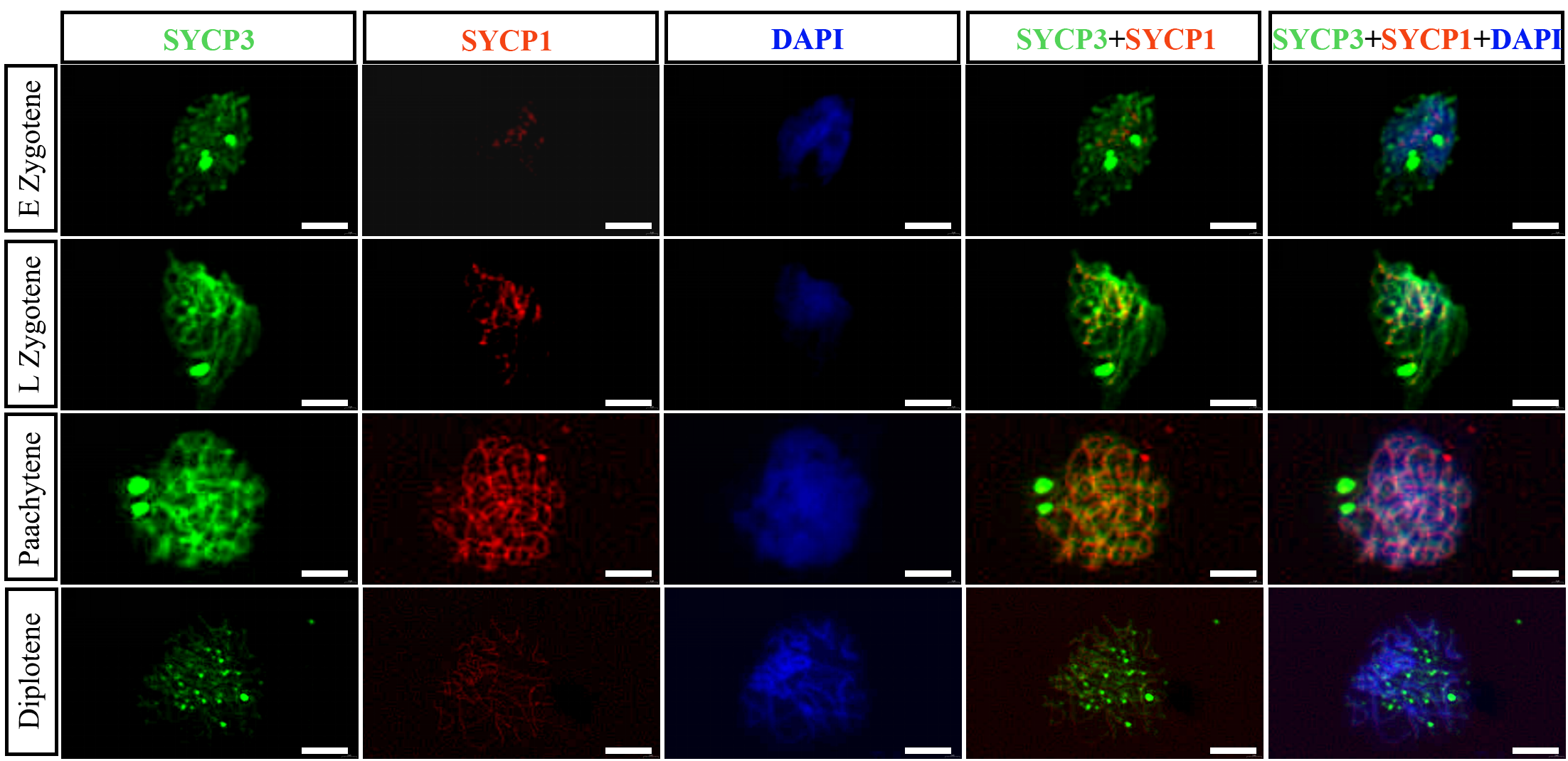

Supplement: Supplementary file 1 [file biology-14-01752-s001.zip › Supplementary Material/Fig. S2.tif]

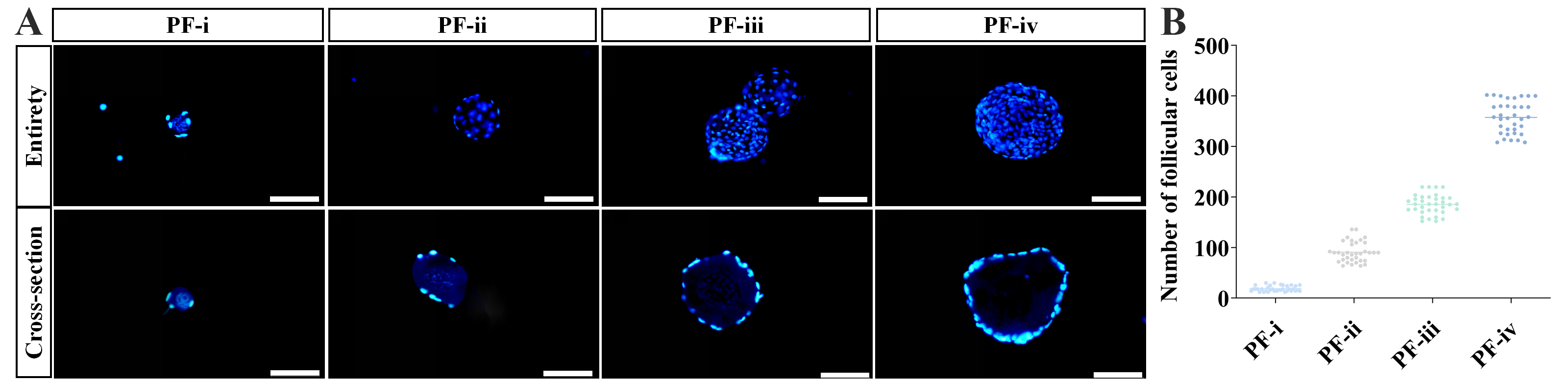

Supplement: Supplementary file 1 [file biology-14-01752-s001.zip › Supplementary Material/Fig. S3.tif]

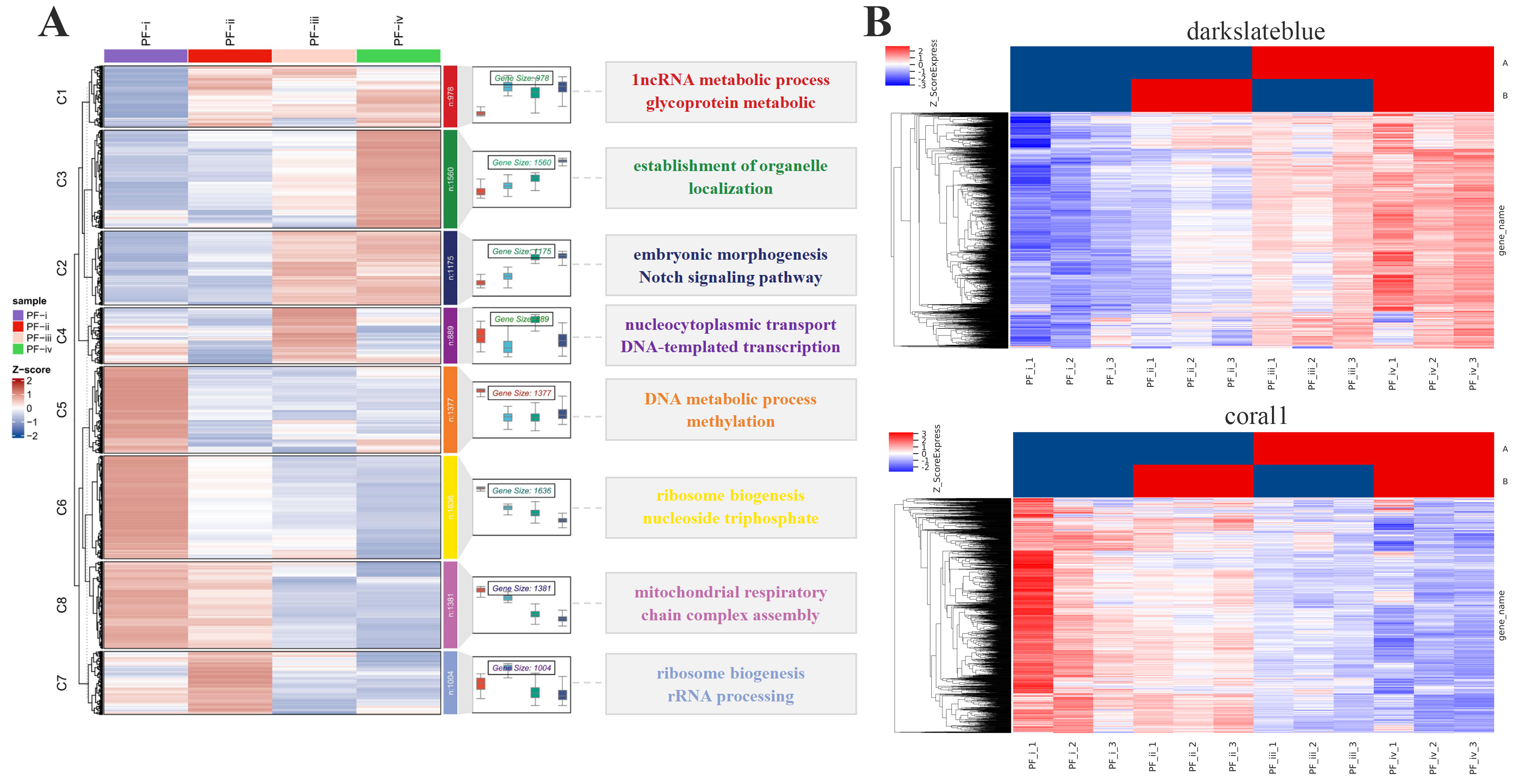

Supplement: Supplementary file 1 [file biology-14-01752-s001.zip › Supplementary Material/Fig. S4.tif]

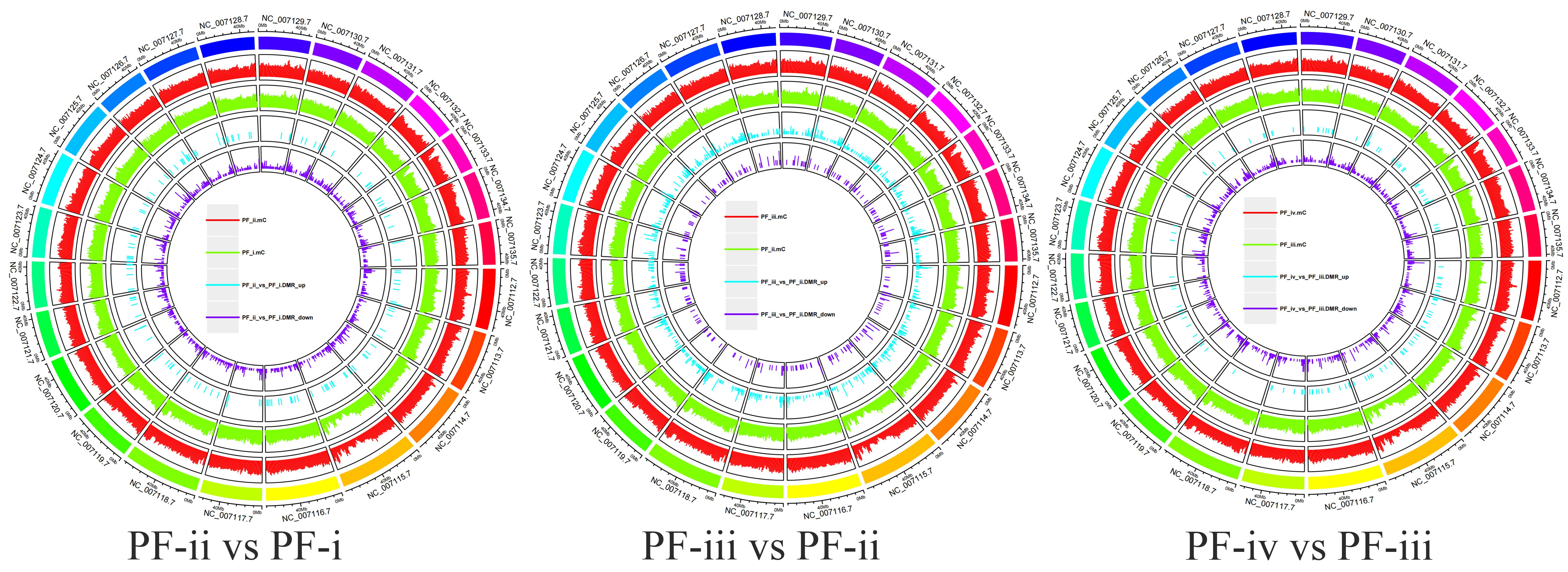

Supplement: Supplementary file 1 [file biology-14-01752-s001.zip › Supplementary Material/Fig. S5.tif]

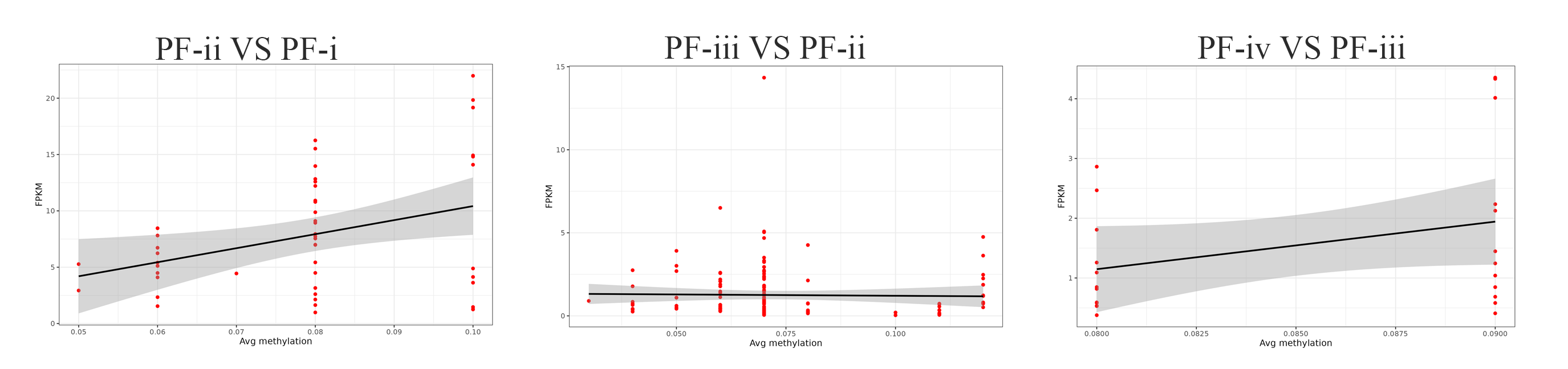

Supplement: Supplementary file 1 [file biology-14-01752-s001.zip › Supplementary Material/Fig. S6.tif]
